# Multipronged SMAD pathway targeting by lipophilic poly(β-amino ester) miR-590-3p nanomiRs inhibits mesenchymal glioblastoma growth and prolongs survival

**DOI:** 10.1038/s41392-025-02223-w

**Published:** 2025-04-30

**Authors:** Jack Korleski, Sophie Sall, Kathryn M. Luly, Maya K. Johnson, Amanda L. Johnson, Harmon Khela, Bachchu Lal, TC Taylor, Jean Micheal Ashby, Hector Alonso, Alice Li, Weiqiang Zhou, Karen Smith-Connor, Russell Hughes, Stephany Y. Tzeng, John Laterra, Jordan J. Green, Hernando Lopez-Bertoni

**Affiliations:** 1 Hugo W. Moser Research Institute at Kennedy Krieger, Baltimore, USA; 2 Department of Internal Medicine, Mayo Clinic Rochester, Minnesota, USA; 3 Department of Biomedical Engineering and the Translational Tissue Engineering Center, Johns Hopkins University School of Medicine, Baltimore, USA; 4 Department of Biology, Johns Hopkins University, Baltimore, USA; 5 Department of Neurology, Johns Hopkins University School of Medicine, Baltimore, USA; 6 Department of Neuroscience, Johns Hopkins University, Baltimore, USA; 7 Department of Biostatistics, Johns Hopkins Bloomberg School of Public Health, Baltimore, USA; 8 Single Cell & Transcriptomics Core at the Institute for Basic Biomedical Sciences, Johns Hopkins University School of Medicine, Baltimore, USA; 9 Department of Neuroscience, Johns Hopkins University School of Medicine, Baltimore, USA; 10 Department of Oncology, Johns Hopkins University School of Medicine, Baltimore, USA; 11 Department of Ophthalmology, Johns Hopkins University School of Medicine, Baltimore, USA; 12 Departments of Materials Science & Engineering and Chemical & Biomolecular Engineering, Johns Hopkins University, Baltimore, USA; 13 Department of Neurosurgery, Johns Hopkins University School of Medicine, Baltimore, USA; 14 Sidney Kimmel Comprehensive Cancer Center at Johns Hopkins, Baltimore, USA

**Keywords:** CNS cancer, Cancer stem cells, Drug development

## Abstract

Despite aggressive therapy, glioblastoma (GBM) recurs in almost all patients and treatment options are very limited. Despite our growing understanding of how cellular transitions associate with relapse in GBM, critical gaps remain in our ability to block these molecular changes and treat recurrent disease. In this study we combine computational biology, forward-thinking understanding of miRNA biology and cutting-edge nucleic acid delivery vehicles to advance targeted therapeutics for GBM. Computational analysis of RNA sequencing from clinical GBM specimens identified TGFβ type II receptor (TGFBR2) as a key player in the mesenchymal transition associated with worse outcome in GBM. Mechanistically, we show that elevated levels of TGFBR2 is conducive to reduced temozolomide (TMZ) sensitivity. This effect is, at least partially, induced by stem-cell driving events coordinated by the reprogramming transcription factors Oct4 and Sox2 that lead to open chromatin states. We show that blocking TGFBR2 via molecular and pharmacological approaches decreases stem cell capacity and sensitivity of clinical recurrent GBM (rGBM) isolates to TMZ in vitro. Network analysis uncovered miR-590-3p as a tumor suppressor that simultaneously inhibits multiple oncogenic nodes downstream of TGFBR2. We also developed novel biodegradable lipophilic poly(β-amino ester) nanoparticles (LiPBAEs) for in vivo microRNA (miRNAs) delivery. Following direct intra-tumoral infusion, these nanomiRs efficiently distribute through the tumors. Importantly, miR-590-3p nanomiRs inhibited the growth and extended survival of mice bearing orthotopic human rGBM xenografts, with an apparent 30% cure rate. These results show that miRNA-based targeted therapeutics provide new opportunities to treat rGBM and bypass the resistance to standard of care therapy.

## Introduction

Transforming growth factor-beta (TGFβ) signaling plays a crucial role in the regulation of many cellular processes, including tumor progression by supporting the stem cell and therapy-resistant phenotype of cells.^1,2^ TGFβ binding triggers TGFBR2 to phosphorylate TGFBR1 and activated TGFBR1 initiates intracellular signaling by phosphorylating SMAD2/3. Phosphorylated SMAD2/3 binds SMAD4 and the SMAD2/3–SMAD4 complex translocates into the nucleus to modify expression of target genes.^3^ Numerous anti-TGFβ strategies have been developed with very promising pre-clinical results but limited clinical efficacy.^3^ These strategies have mainly focused on understanding and targeting TGFβ and TGFBR1 with little attention paid TGFBR2 function. Emerging evidence suggests TGFBR2 signaling can support oncogenic mechanisms in glioma cells, including resistance to therapy and maintenance of the stem cell phenotype,^4–8^ and blocking these signaling in gliomas may offer therapeutic advantages in the context of recurrent disease.^4,7^

Glioblastoma (GBM) is the most aggressive and common form of primary central nervous system (CNS) malignancies.^9^ Despite very aggressive treatment,^10^ less than 10% of GBM patients survive more than five years after initial diagnosis and the median survival time after recurrence is only 6-9 months. A major challenge in developing effective anti-GBM therapies is the dynamic and heterogeneous nature of the disease.^11,12^ This high-degree of adaptability is thought to influence the emergence of therapy-resistant GBM cell sub-populations,^13^ with stem-like cell subsets playing a major role in the process.^14,15^ The mesenchymal subtype, thought to be the most aggressive, is highly associated with recurrent disease in GBM.^16,17^ It is therefore imperative that we understand the molecular mechanisms underlying GBM cell plasticity to design rational therapeutics that eliminate transitions between cellular states to reduce the emergence of therapy-resistant cells.

miRNAs are small, non-coding RNA molecules that have important functions in regulating gene expression by targeting messenger RNAs (mRNAs) for degradation or translational repression. There is rich cross-talk between miRNAs and TGFβ signaling comprising of feedback regulation.^18,19^ As a result, miRNAs have emerged as promising candidates for anti-cancer therapeutics blocking TGFβ signaling. Advances in nucleic acid delivery modalities now allow us to combine this understanding with the promiscuous nature of miRNAs to inhibit oncogenic networks.^20^ Revisiting classical oncogenic pathways, such as TGFβ signaling, with new mechanistic insights and tools can yield novel therapeutics to improve GBM patient outcomes.

The goal of this study is to advance the development of miRNA-based therapeutics targeting the stem cell phenotype of GBM cells and their capacity to establish and maintain recurrent disease. We identified a unique role for TGFBR2 signaling as a target in rGBM cells. We show miR-590-3p functions as a tumor suppressor that promiscuously blocks multiple SMAD2/3 targets downstream of TGFBR2 in GBM neurospheres. We developed next-generation LiPBAE-based nanoparticles (NPs) for in vivo miRNA delivery to established orthotopic models of rGBM. Following direct intra-tumoral infusion, these miRNA-containing NPs (nanomiRs) were found to efficiently distribute through tumors, inhibit the growth of pre-established orthotopic human rGBM xenografts in mice and prolong survival with an ~30% cure rate. These results show that the promiscuous nature of miRNAs, in combination with cutting-edge nucleic acid delivery vehicles, can be leveraged as advanced targeted therapeutics for rGBM.

## Results

### Stem-cell driving mechanisms affect temozolomide sensitivity of GBM cells by directly activating TGFBR2 expression

**Fig. 1:**
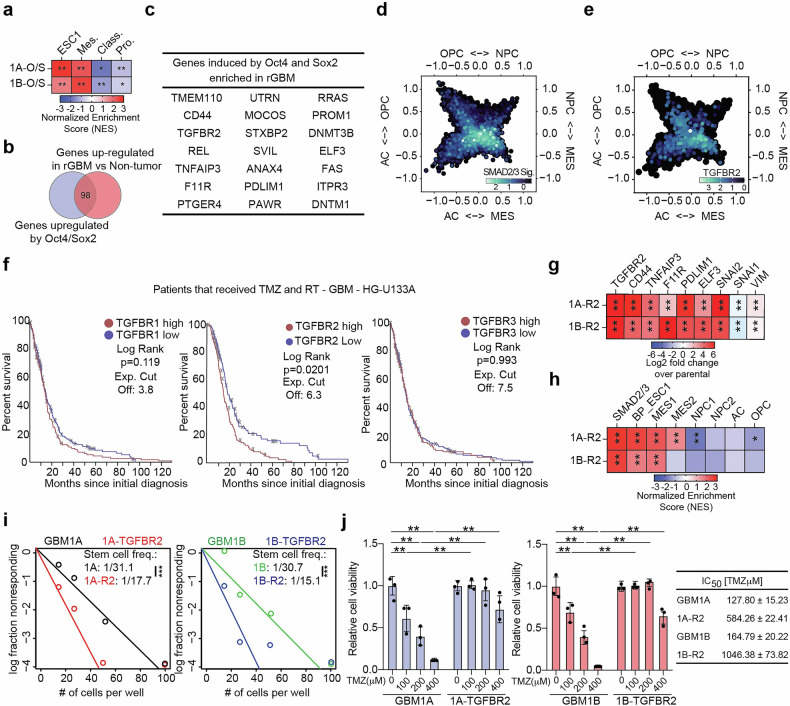
TGFBR2 signaling associates with the therapy-resistant state in GBM. **a** Heatmap showing normalized enrichment scores (NES) from GSEA of gene signatures GBM subtypes. **b** Venn diagram showing genes induced by Oct4 and Sox2 in GBM neurospheres and also upregulated in rGBM compared to non-tumor tissue. **c** Table summarizing statistically significant genes upregulated in GBM neurospheres expressing transgenic Oct4 and Sox2 and rGBM. Two-dimensional butterfly plot visualization of snRNA-Seq data generated from rGBM clinical specimens (5 male and 5 female datasets; GSE174554) based on molecular subtype signature scores determined by Neftel et al. showing (**d**) SMAD2/3 target gene signatures and (**e**) TGFBR2 expression. **f** Kaplan-Meier survival curve of GBM patients with high vs. low TGFBR2 expression that received an aggressive course of therapy. **g** Heatmap showing expression of selected genes from RNA-Seq performed in GBM neurospheres expressing transgenic TGFBR2. **h** Heatmap showing normalized enrichment scores (NES) from GSEA of gene signatures associated with the transition to a therapy-resistant state in GBM. **i** Equal numbers of GBM neurospheres with (1A-R2 or 1B-R2) and without (1A or 1B) transgenic TGFBR2 were seeded under limiting dilution conditions and stem cell frequency was determined after 14 days. **j** Equal numbers of GBM neurospheres with and without transgenic TGFBR2 were treated with TMZ and cell viability was measured 5 days after treatment via Cell Titer Glo assay. Statistical significance was calculated using unpaired, non-parametric, student T-test with Mann–Whitney post hoc test in panel **f**; Student T-test was used to determine statistical differences in panels **h** and **i**; One-way ANOVA with Tukey’s post hoc test was used to calculate statistical significance in panel **j**. Data are presented as means±S.D. *p < 0.05; **p < 0.01; ***p < 0.001

As mentioned earlier, the stem cell phenotype of GBM cells is a critical determinant of tumor progression and therapy resistance.^14^ Oct4 and Sox2 drive GBM cell transition to a stem-like tumor-propagating state characterized by enhanced stemness and resistance to standard-of-care treatments.^15^ To define events that contribute to therapy resistance downstream of Oct4 and Sox2 in GBM we performed RNA sequencing (RNA-seq) on patient-derived neurospheres (i.e. GBM1A and GBM1B) with and without transgenic co-expression of Oct4 and Sox2 (i.e. GBM1A-O/S and GBM1B-O/S) (supplementary data set 1). Genes commonly upregulated by co-expressing Oct4 and Sox2 in 2 distinct patient-derived neurosphere lines (log2 > 1; *p* < 0.05) were cross-referenced to transcriptomic signatures related to cellular transitions associated with patient outcome in GBM^16,21^ (i.e. classical, mesenchymal and proneural). This analysis shows these cells are enriched in transcriptomic signatures associated with mesenchymal and embryonic stem cells (Fig. 1a), thought to be the most aggressive cell subsets and critical contributors of therapeutic resistance.^16,17^ Next, we performed a similar analysis comparing transcripts differentially regulated in GBM patients upon recurrence (i.e. second resection and after receiving therapy) and non-tumor brain tissue and compared this gene list to the transcripts induced by Oct4 and Sox2 in our patient-derived neurospheres. This comparison identified 98 genes consistently up-regulated (log2 fold change >1), 21 of which met statistical significance parameters of p < 0.05, in both specimens from rGBM and neurospheres expressing transgenic Oct4/Sox2 (Fig. 1b, c). Interestingly, 6 of the 21 genes identified (CD44, TGFBR2, TNFAIP3, F11R, PDLIM1, and ELF3) regulate oncogenic properties and belong to the TGFβ signaling axis, including signal initiator TGFBR2^6,22–27^ (Fig. 1c, **genes in bold**).

To further explore the clinical relevance of these observations, we analyzed single-cell RNA-Seq (scRNA-Seq) datasets from rGBM patient specimens and found that TGFBR2 and SMAD2/3 target gene signatures are broadly expressed in this cell population, with the highest expression observed in mesenchymal-like (Mes-like) GBM cell subsets (Fig. 1d, e). Complimentary analyses performed in scRNA-Seq datasets from patient-derived glioma stem cells (GSCs) found similar trends (Supplementary Fig. 1a). We also measured a positive correlation between mesenchymal marker CD44, SMAD2/3 targets, and gene signatures enriched in Mes-like GBM cells and TGFBR2 expression (supplementary Fig. 1b). Interestingly, we observe weaker correlations between these gene signatures and TGFBR1 and TGFBR3, suggesting a distinct role for TGFBR2 signaling in these cell subsets. Further dissection of clinical transcriptomic datasets shows no increase in TGFBR2 expression in samples derived from GBM patients upon first (treatment naïve) or second (post-therapy) resection (Supplementary Fig. 2a), however, TGFBR2 transcripts are consistently upregulated in clinical GBM specimens of the mesenchymal subtype (Supplementary Fig. 2b). Additional analysis shows that patients with lower levels of TGFBR2 have better outcomes after receiving aggressive course of therapy comprised of both TMZ and radiation (Fig. 1f). Notably, while TGFBR1 levels are also increased in patient specimens of the mesenchymal subtype, this is without an associated survival difference (Fig. 1f and Supplementary Fig. 2). These observations are consistent high levels of TGFBR2 driving a mesenchymal transition in GBM stem-like cells and playing an important role in controlling sensitivity to temozolomide (TMZ).

To test the functional relevance of these associations, we expressed transgenic TGFBR2 in two distinct GBM neurosphere isolates via lentiviral delivery systems and performed unbiased transcriptomic analysis by RNA-Seq (Supplementary dataset 2). These cell models express comparable levels of transgenic TGFBR2 and high levels of SMAD2/3 targets compared to the parental controls, demonstrating SMAD signaling activation (Fig. 1g). Gene set enrichment analysis (GSEA) of these transcriptomic data sets shows enrichment of gene signatures indicative of a transition to a more mesenchymal and stem-like state, salient features of clinical rGBM^28,29^ (Fig. 1h). Consistent with these transcriptomic and clinical associations, neurospheres expressing transgenic TGFBR2 gain self-renewal capacity (Fig. 1i) and become less sensitive to TMZ (Fig. 1j). Taken together, these data are consistent with TGFBR2 activating stemness-driving reprogramming events and signaling through TGFBR2 playing an important role in determining the sensitivity of GBM cells to TMZ.

**Fig. 2:**
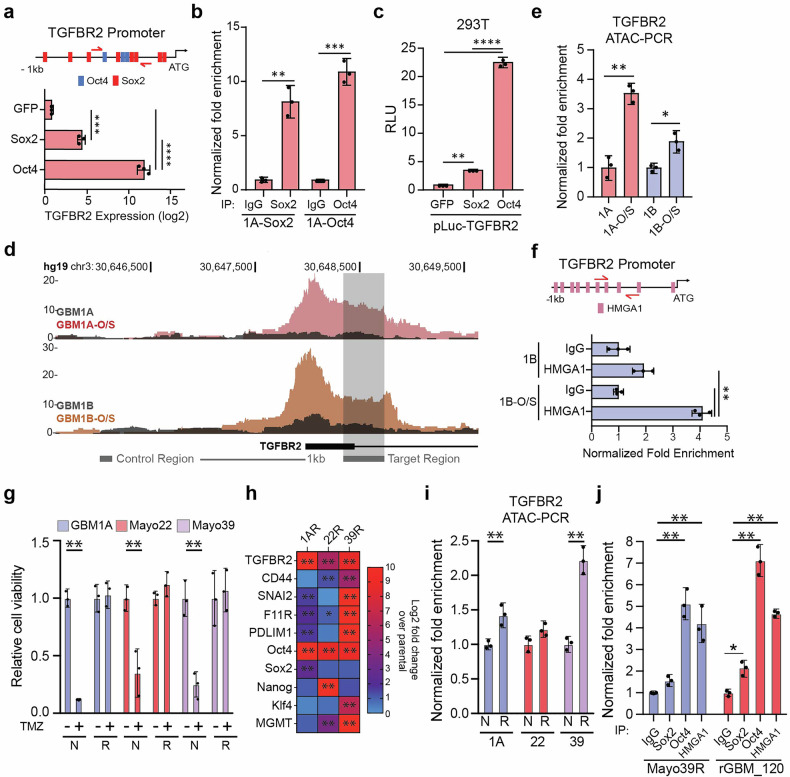
Changes in chromatin architecture activates TGFBR2 expression in TMZ-insensitive GBM neurospheres. **a** Schematic showing predicted binding sites for Oct4 (blue) or Sox2 (red) on the TGFBR2 promoter. Red arrows mark the primer sites used for down-stream analysis (top panel). qRT-PCR analysis showing TGFBR2 expression in GSCs expressing transgenic Sox2 or Oct4. GFP was used as a negative control. **b** DNA purified from chromatin immuno-precipitation was analyzed by qRT-PCR using primer pairs designed to amplify fragments containing Sox2 and Oct4 binding sites shown in (**a**). **c** 293 T cells were co-transfected with a luciferase reporter construct spanning the TGFBR2 putative promoter containing the Sox2 and Oct4 binding sites and GFP, Oct4 or Sox2 and luciferase activity was measured 2 days after transfection. **d** ATAC-Seq tracks showing chromatin accessibility in GSCs with and without transgenic Oct4/Sox2 expression. Grey boxes mark priming sites used for downstream analysis. **e** DNA purified from chromatin that underwent Tn5 transposition was analyzed by qRT-PCR using primer pairs designed to amplify fragments predicted to reside in euchromatin regions spanning the TGFBR2 promoter. **f** Schematic showing predicted HMGA1 binding site to the TGFBR2 promotor (top panel) and ChIP-PCR showing architectural transcription factor HMGA1 bind to TGFBR2 promoter. **g** GBM neurospheres were exposed to 2 Gy of ionizing radiation and 200 μM of TMZ and surviving clones were expanded over several weeks. Equal numbers of naïve (N) and therapy-surviving (R) cells were treated with TMZ and cell viability was measured 5 days after treatment via Cell Titer Glo assay. **h** qRT-PCR analysis showing expression of TGFBR2, mesenchymal marker CD44, SMAD target genes (SNAI2, F11R, and PDLIM1), stem cell drivers (Oct4, Sox2, Kl4, and Nanog), and DNA repair enzyme MGMT in naïve and therapy-surviving GBM neurosphere pairs. **i** DNA purified from chromatin that underwent Tn5 transposition in naïve and resistant GBM neurospheres was analyzed by qRT-PCR using primers spanning the TGFBR2 promoter. **j** DNA purified from chromatin immuno-precipitation in TMZ resistant GBM neurospheres (Mayo 39 R) or rGBM (GBM_120) clinical specimens was analyzed by qRT-PCR using primer pairs designed to amplify fragments containing Sox2 and Oct4 binding sites shown in (**a**). Student T-test was used to determine statistical differences in panels **b, e, f** and **i**; One-way ANOVA with Tukey’s post hoc test was used to calculate statistical significance in panel **a, c, g** and **j**. Data are presented as means±S.D. *p < 0.05; **p < 0.01; ***p < 0.001; ****p < 0.0001

To dissect how drivers of stemness coordinate TMZ sensitivity of GBM cells induced by TGFBR2, we queried the TGFBR2 promoter and identified several Oct4 and Sox2 binding sites within 1 kb of the translation start site (Fig. 2a, top panel and Supplementary Fig. 3). Consistent with the presence of these binding sites, transgenic expression of either Oct4 or Sox2, but not green fluorescent protein (GFP), was sufficient to induce TGFBR2 transcripts in GBM neurospheres (Fig. 2a, bottom panel). Chromatin immunoprecipitation (ChIP) analysis of a subset of putative binding sites confirmed Sox2 and Oct4 binding to the TGFBR2 promoter (Fig. 2b), consistent with the direct regulation of TGFBR2 expression by Sox2 and Oct4 in GBM neurospheres. Exogenous Sox2 or Oct4 specifically induced luciferase activity from a reporter containing the TGFBR2 promoter region encompassing the Sox2 and Oct4 binding sites identified in 1a (Fig. 2c). We recently reported that the architectural transcription factor high mobility group protein A1 (HMGA1) plays a critical role in euchromatin formation and gene expression changes during the epigenetic reprogramming of GBM cells to a stem-like state.^30^ Assay for transposase-accessible chromatin with sequencing (ATAC-Seq) experiments show increased chromatin accessibility in regions overlapping exon 1 of TGFBR2 in GBM neurospheres expressing transgenic Sox2 and Oct4 compared to parental controls (Fig. 2d). Our unbiased sequencing results were confirmed by ATAC followed by quantitative reverse transcription polymerase chain reaction (qRT-PCR) using primers spanning the predicted open chromatin regions (Fig. 2e). We also found that HMGA1 is enriched in the TGFBR2 promoter region containing the Oct4 and Sox2 binding sites, consistent with a euchromatin transition (Fig. 2f and supplementary Fig 2).

To determine if these chromatin transitions occur in clinical GBM specimens as they lose ionizing radiation (IR) and TMZ sensitivity, we treated patient-derived GBM cells with IR and TMZ and expanded resistant surviving clones (GBM1AR, Mayo22R, and Mayo39R) (Fig. 2g). The cell subsets emerging from this treatment consistently expressed higher levels of the pluripotency factor Oct4 and TGFBR2 (Fig. 2h). Interestingly, only resistant clones derived from Mayo39 and GBM1A cells expressed higher levels of SMAD2/3 targets (CD44, SNAI2, F11R, and PDLIM1) (Fig. 2h). Additionally, we only observed an increase in O-6-Methylguanine-DNA Methyltransferase (MGMT) transcript levels in Mayo22R and Mayo39R, highlighting the high degree of heterogeneity among cells surviving standard-of-care therapy. ATAC-PCR analysis shows this increase in TGFBR2 expression is concurrent with an increase in chromatin accessibility in GBM1AR and Mayo39R, consistent with SMDA2/3 target upregulation (Fig. 2h, i). Consistent with these findings from experimental cell models in vitro, ChIP-PCR analysis shows Sox2, Oct4, and HMGA1 binding to the TGBFR2 promoter in cells isolated from recurrent GBM patient-derived xenografts (PDX) (Fig. 2j). Taken together, these results demonstrate that TGFBR2 expression can be induced by reprogramming transcription factors and chromatin remodeling events associated with decreased TMZ sensitivity in GBM cells.

### TGFBR2 signaling regulates TMZ sensitivity of rGBM cells

**Fig. 3:**
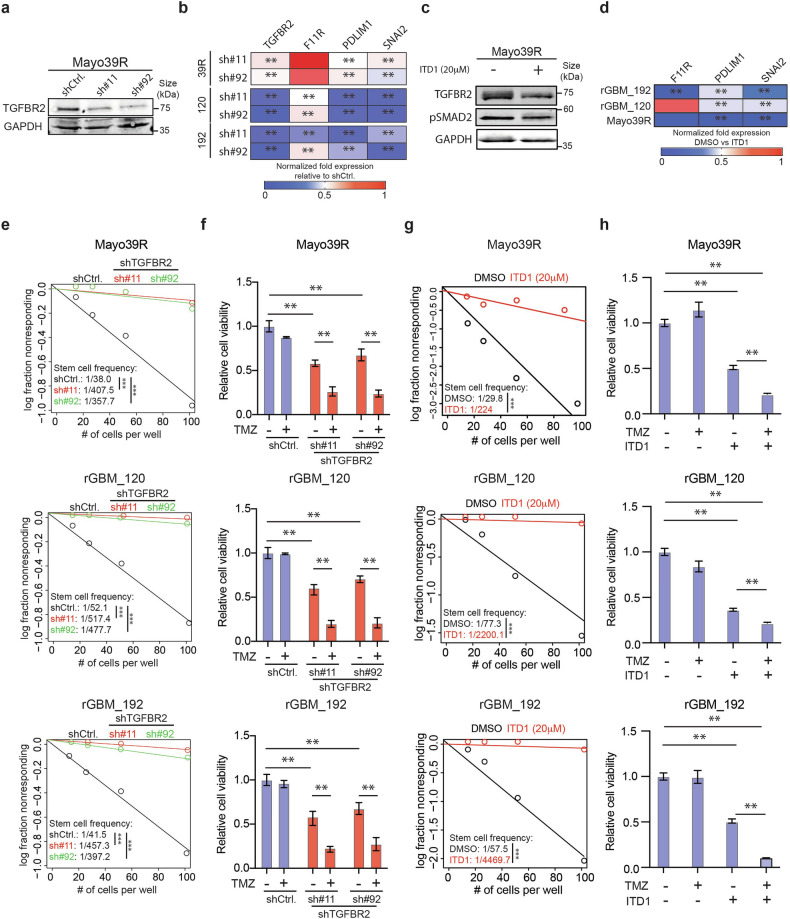
TGFBR2 inhibition increases sensitivity of GBM neurospheres to TMZ. GBM neurospheres were transduced with 2 independent shRNAs and expression of TGFBR2 was measured after 5 days via western blot (**a**) or qRT-PCR (**b**). **c** Western blot showing expression of TGFBR2 and pSMAD2 48 h after ITD1 treatment in GBM neurospheres. **d** qRT-PCR analysis showing expression of down-stream TGFβ targets 5 days after ITD1 treatment in GBM neurospheres. **e** ELDA assay to measure stem cell frequency 14 days after TGFBR2 knock-down in GBM neurospheres. **f** GBM neurospheres received TMZ (200 μM) 3 days after transduction with lentivirus expressing 2 independent shRNAs against TGFBR2. Cell viability was measured 2 days after TMZ treatment using CTG assay. **g** ELDA assay to measure stem cell frequency 14 days after ITD1 treatment in GBM neurospheres and clinical rGBM isolates. **h** Therapy-resistant and clinical rGBM specimens were treated with ITD1 (20 μM), TMZ (200 μM), or the combination and cell viability was measured using CTG assay 5 days after treatment. Student T-test was used to determine statistical differences in panels **b** and **d**. One-way ANOVA with Tukey’s post hoc test was used to calculate statistical significance in panel **f**, **h**. Data are presented as means±S.D. **p < 0.01; ***p < 0.001

**Fig. 4:**
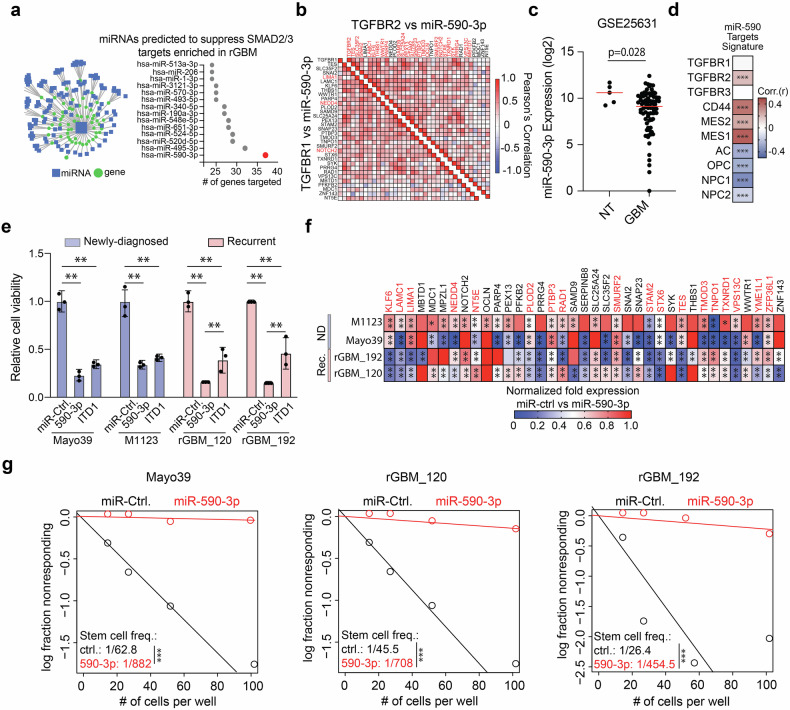
miRNA-based targeting of TGFBR2 pathway inhibits the stem cell phenotype rGBM cells. **a** Network of miRNA and gene targets (left panel), Number of predicted SMAD2/3 targets enriched in rGBM by miRNA (right panel). **b** Gene expression correlation analysis between TGFBR2 or TGFBR1 and the predicted miR-590-3p targets in rGBM clinical specimens (HG-U133A). Genes highlighted in red show statistically significant positive correlations. **c** Expression of miR-590-3p in GBM clinical specimens compared to Non-tumor tissue. **d** Pearson’s correlation coefficient for gene signature composed of miR-590-3p targets with TGFBR1, TGFBR2, TGFBR3 expression and markers or gene signatures related to GBM cell subsets determined from scRNA-Seq derived from GSCs. **e** Cell viability 5 days after miR-590-3p or ITD1 treatment. **f** Expression of miR-590-3p predicted targets by qRT-PCR 5 days after transgenic miR-590-3p expression. **g** ELDA assay measuring stem cell frequency 14 days after transgenic miR-590-3p expression in rGBM cells. Statistical significance was calculated using unpaired, non-parametric, student T-test with Mann–Whitney post hoc test in panel **c**; Student T-test was used to determine statistical differences in panel **f**; One-way ANOVA with Tukey’s post hoc test was used to calculate statistical significance in panels **d** and **e**. Data are presented as means±S.D. **p < 0.01; ***p < 0.001

Our results thus far predict that blocking TGFBR2 will inhibit the stem cell phenotype and decrease the sensitivity of GBM neurospheres to TMZ. To test this hypothesis, we used two independent shRNAs (Fig. 3a, b) and ITD1^31^ (Fig. 3c, d), a specific TGFBR2 small-molecule inhibitor, to block TGFBR2 signaling in GBM neurospheres. Consistent with our predictions, inhibiting TGFBR2 blocked SMAD2/3 signaling in TMZ-insensitive GBM cells, decreased self-renewal capacity (Fig. 3e, g) and restored sensitivity to TMZ treatment (Fig. 3f, h). As mentioned earlier, substantial efforts have been made to develop strategies to block TGFβ signaling in cancer, including anti-sense oligonucleotides (ASOs) and small-molecule inhibitors, resulting in limited clinical efficacy.^3^ These shortcomings are attributed to poor pharmacodynamics, resistance due to compensatory mechanisms, off-target effects, and dose-limiting systemic toxicity.^3^ Novel strategies that directly block multiple nodes within the TGFβ signaling pathway have the potential to overcome these limitations. Toward this end, we looked for miRNAs that can target multiple genes within the subset of SMAD2/3 targets enriched in rGBM. This analysis predicted miR-590-3p would inhibit 37 SMAD 2/3 gene targets enriched in rGBM (Fig. 4a). Computational analysis also showed a positive correlation between TGFBR2 expression and 24 of the 37 miR-590-3p targets in rGBM clinical specimens (Fig. 4b). Interestingly, only 3 of the 37 targets show a statistically significant positive correlation with TGFBR1 in the same patient specimens (Fig. 4b). Additionally, miR-590-3p expression is decreased in GBM clinical specimens compared to non-tumor tissue, suggesting a tumor-suppressive function (Fig. 4c). We also measured a strong correlation between these 37 genes and TGFBR2, CD44 and Mes-like signatures in scRNA-Seq datasets derived from GSCs (Fig. 4d and supplementary Fig 1a). Consistent with these predictions, transgenic expression of miR-590-3p robustly inhibited cell proliferation capacity of Mes-like newly diagnosed (Mayo39 and M1123) and rGBM (rGBM_120 and rGBM_192) neurospheres compared to a control miRNA predicted to have limited capacity to inhibit TGFBR2 targets (Fig. 4e). miR-590-3p also consistently inhibited expression of 18 of the 37 targets across 2 distinct newly diagnosed and 2 distinct rGBM neurosphere models (Fig. 4f) concurrent with a robust inhibition of self-renewal capacity (Fig. 4g and Supplemental Fig. 4). Taken together these results show that miR-590-3p is a potent inhibitor of SMAD2/3 signaling in Mes-like GBM cells and predicts significant anti-tumor effects in rGBM models.

### Biodegradable LiPBAE encapsulates and effectively delivers miRNAs to human rGBM in vitro and in vivo

**Fig. 5:**
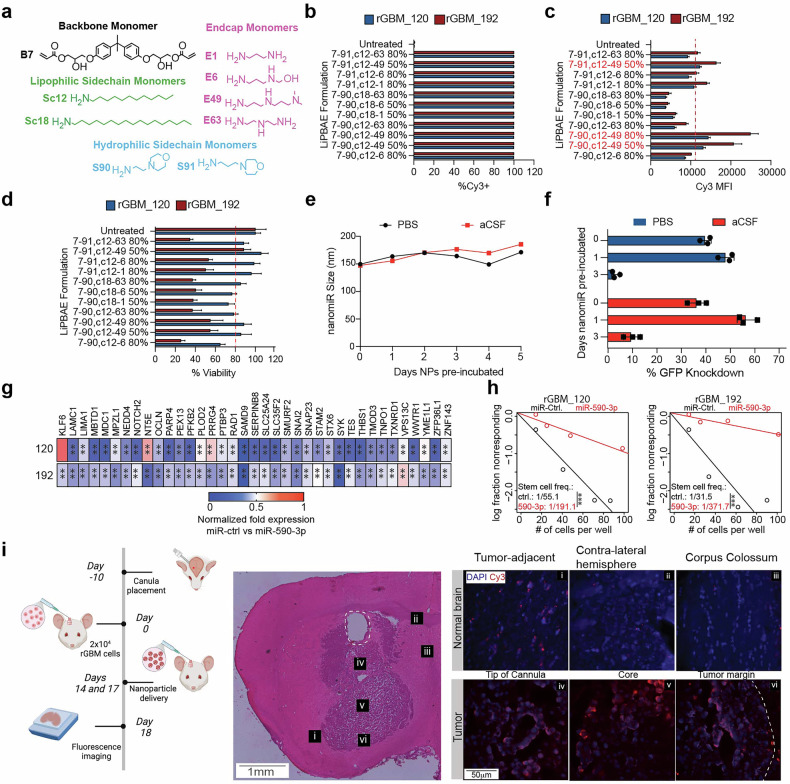
Novel LiPBAE has low toxicity and effectively delivers miRNAs to rGBM cells in vitro and in vivo. **a** LiPBAEs were synthesized using structurally diverse monomers. Each LiPBAE is composed of the B7 backbone monomer, a blend of hydrophilic sidechain and lipophilic sidechain monomers (% lipophilic sidechain monomer indicated), and an endcap monomer. Uptake efficacy in rGBM_120 and rGBM_192 cells 24 h following nanomiR administration represented as %Cy3+ cells (**b**) and Cy3 mean fluorescence intensity (MFI) (**c**). **d** Cell viability was assessed via cell titer glo 24 h following nanomiR administration. **e** PEGylated 7-90,c12-49 80% nanomiRs maintained their small size after up to five days of incubation in PBS or artificial CSF (aCSF). **f** PEGylated 7–90,c12–49 80% nanomiRs enabled functional delivery of siGFP in vitro following 24 h of incubation in PBS or aCSF. **g** qRT-PCR analysis showing expression of miR-590-3p targets 5 days after nanomiR transfections in rGBM clinical specimens. **h** ELDA assay to measure stem cell frequency 14 days after nanomiR transfection in rGBM clinical specimens. **i** Schematic showing in vivo PEGylated nanomiR delivery using fluorescently-tagged control miRNA (top left panel). H&E-stained section showing established orthotopic rGBM (bottom left panel). Dotted lines outline canula track and boxes mark regions used to capture fluorescence images. Fluorescence imaging showing Cy3 signal within the tumor and in different parts of the brain (bottom right panel). Student T-test was used to determine statistical differences in panel **g**. Data are presented as means±S.D. **p < 0.01. ***p < 0.001

To extend these initial results towards in vivo applications, a library of biodegradable LiPBAEs was synthesized using structurally diverse monomers (Fig. 5a and supplementary Fig 5). To assess delivery to rGBM, LiPBAEs were allowed to self-assemble with Cyanine3 (Cy3)-labeled miRNA, and the resulting nanomiRs were administered to rGBM_120 and rGBM_192 cells. Uptake analysis via flow cytometry revealed robust uptake of the nanomiRs (Fig. 5b, c), with varying degrees of cellular toxicity across nanomiR formulations (Fig. 5d). To confirm functional oligo delivery with LiPBAEs, the top formulations that demonstrated highest uptake with moderate cellular toxicity were then assessed and LiPBAE 7-90,c12-49 80% (Fig. 5b, c) was chosen as the lead polymer for all subsequent studies. As PEGylation is a common strategy to improve nanoparticle stability and efficacy in vivo,^32–34^ we next assessed PEGylated LiPBAE nanomiR stability and efficacy in vitro. To determine downstream applicability of LiPBAE 7-90,c12-49 80% for in vivo application, PEGylated nanomiRs were pre-incubated in PBS and artificial cerebrospinal fluid (aCSF) at 37 °C for 0 to 5 days. Dynamic light scattering (DLS) indicated consistent nanomiR size over 5 days (Fig. 5e), and though nanomiR function decreased by day 3, efficacy remained high after incubation for 24 h in PBS or aCSF (Fig. 5f), suggesting that the PEGylated nanomiRs could maintain their physical properties and efficacy after administration in vivo.

To test the efficacy of LiPBAE nanomiRs, rGBM cells were transfected using nanomiRs generated by complexing a control miRNA or miR-590-3p using 7–90,c12–49 80%. As expected, nanomiR transfection of miR-590-3p robustly decreased target expression (Fig. 5g) and self-renewal capacity of two distinct rGBM PDX cell lines (Fig. 5h). Notably, miR-590-3p delivery using polymer 7-90,c12-49 80% inhibited target expression more comprehensively than commercially available delivery vehicles– 36 of 37 compared to 25 of 37 targets (Fig. 4f, g). Direct intratumoral delivery of miRNA via 7–90,c12–49 80%-based PEGylated nanomiRs was used to assess miRNA biodistribution and in vivo bioactivity. Fourteen days after cell implantation, animals received 2 infusions of PEGylated nanomiRs containing Cy3-labeled control miRNA. Cy3-labled miRNA was found to be distributed throughout the tumor when evaluated 4 days after the first nanomiR infusion and 1 day after the last infusion. Cy3 signal was also found for at least 200 mm adjacent to the tumor margins, suggesting that the PEGylated nanomiRs can reach invading tumor cells spreading beyond identifiable tumor margins (Fig. 5i).

### miRNA delivery via PEGylated biodegradable LiPBAE nanomiRs inhibits tumor growth and prolongs animal survival in an orthotopic model of human rGBM

**Fig. 6:**
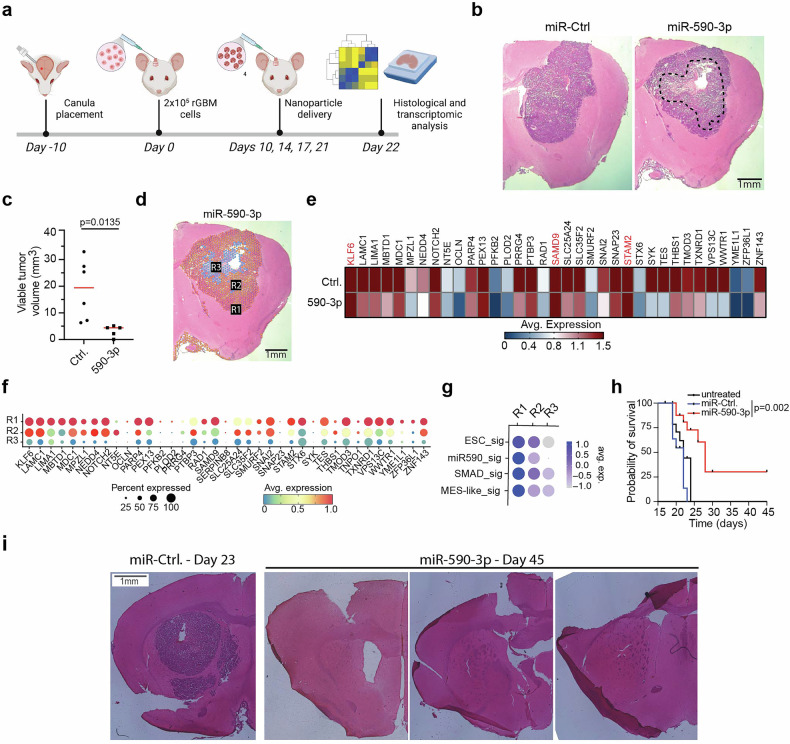
In vivo delivery of PEGylated miR-590-3p nanomiRs inhibits rGBM growth and extends survival in orthotopic rGBM mouse models. **a** Schematic showing the in vivo delivery of PEGylated miR-590-3p nanomiRs. **b** H&E-stained brain sections from mice bearing orthotopic rGBM 22 days after receiving PEGylated nanomiRs loaded with control miRNA or miR-590-3p. Dotted line outlines necrotic region. **c** Viable tumor was measured from H&E-stained sections using computer assisted image analysis. **d** Spatial transcriptomic analysis identified 3 distinct regions in tumors treated with PEGylated miR-590-3p nanomiRs. **e** Transcriptomic analysis of miR-590-3p targets in mice that received PEGylated control miRNA or miR-590-3p nanomiRs. Genes highlighted in red are ones that did not decrease with miR-590-3p treatment. **f** Transcriptomic analysis looking at expression of miR-590-3p targets in mice that received PEGylated miR-590-3p nanomiRs. **g** GSEA from transcriptomic analysis of tumors treated with PEGylated miR-590-3p nanomiRs looking at gene signatures associated with therapy resistance in GBM in the three distinct regions defined in (**d**). **h** Kaplan-Meier survival curve comparing animals that received control miRNA, miR-590-3p or no treatment. Therapy in the survival study was initiated 7 days after tumor cell implantation. Survival was compared across arms using the log-rank test (*N* = 10). **i** H&E-stained sections showing the last animal in the control group and the 3 long-term survivors. Statistical significance was calculated using unpaired, non-parametric, student T-test with Mann–Whitney post hoc test in panel **c**

Our results thus far (Figs. 3–5) support the hypothesis that miR-590-3p delivery using our novel biodegradable LiPBAE polymeric nanomiRs will robustly inhibit growth of rGBM xenografts in vivo. To evaluate the anti-tumor effects of TGFBR2:SMAD targeting via miR-590-3p nanomiR therapy, human rGBM derived PDX cells were implanted following a similar protocol to the one described above (Fig. 6a). We observed a significant decrease in tumor burden in animals treated with active miR-590-3p compared to animals receiving a control miRNA, as determined by computer-assisted morphometry from brains collected after 22 days of treatment (Fig. 6b, c). Spatial transcriptomic analysis (STA) found a decrease in 34 out of the 37 miR-590-3p targets in animals that received the PEGylated nanomiRs delivering miR-590-3p compared to control miRNA (Fig. 6d, e). Furthermore, STA identified 3 cell populations with distinct transcriptomic profiles reflecting a gradient pharmacodynamic response within tumors that received PEGylated miR-590-3p nanomiRs with decreasing target expression towards the core of the tumor closest to catheter tip placement (Fig. 6d, f). We also observed a loss of gene signatures typically associated with therapy-resistance in GBM,^28,29^ including embryonic stem cell (ESC), SMAD signaling, and Mes-like GBM subtype gene signatures towards the core of the tumor (Fig. 6g). To test the effect of miR-590-3p nanomiR therapy on survival, either the control or miR-590-3p nanomiRs were delivered every other day beginning on post-implantation day 7 to animals bearing pre-established orthotopic human rGBM xenografts. All 10 animals that received PEGylated control nanomiRs required euthanasia 23 days post cell implantation. In contrast, 3 of 10 animals treated with miR-590-3p nanomiRs remained alive and healthy until the experiment was ended at day 45 post-implantation (Fig. 6h). Histological analysis of the surviving animals euthanized 45 days post cell implantation revealed that all 3 had no detectable tumor (Fig. 6i). Animals receiving PEGylated miR-590-3p nanomiR therapy began to show signs of weight loss, so treatment was stopped after 7 days (3 injections). All treated animals regained weight within a week after ending treatment. These data demonstrate that miR-590-3p nanomiRs robustly inhibit multiple oncogenic nodes downstream of TGFBR2 to achieve robust anti-tumor effects in an orthotopic mouse model of human rGBM.

## Discussion

Despite aggressive treatment GBM recurrence is essentially inevitable.^10^ Substantial efforts have been dedicated to characterizing the molecular background of GBM cells to identify and block mechanisms conducive to therapy resistance.^11,28,29,35,36^ Initial studies based on bulk gene expression profiling called attention to mesenchymal transitions as critical intermediates of therapy-resistance leading to recurrence in GBM.^16,36^ Interestingly, bulk RNA-sequencing comparing gene expression profiles of GBM samples obtained from patients at initial diagnosis and upon recurrence did not find that recurrent tumors are more mesenchymal than the therapy naïve ones.^35^ More recently, studies using single-cell transcriptomics elegantly highlight the existence of a mesenchymal-like GBM cell subset that strongly associates with aggressive tumor cell phenotypes and recurrence in GBM.^11,28,29^ Single-cell transcriptomic studies from GBM samples obtained from patients at initial diagnosis and upon recurrence convincingly show that therapy induces mesenchymal transitions and activation of stemness-driving events in GBM cells,^28,29^ highlighting the importance of blocking the basic mechanism conducive to these conversions.

TGFβ signaling is a potent driver of cancer progression via the induction of a mesenchymal tumor cell phenotype characterized by enhanced motility, therapeutic resistance, and stem-like properties across tumor types.^1,2^ Tumors carrying a mesenchymal phenotype are thought to respond better to anti-TGFβ therapies.^37–40^ Consistent with previous findings that TGFBR2 signaling can support oncogenic mechanisms in glioma cells,^4–8^ we show that transgenic expression of TGFBR2 is sufficient to drive a mesenchymal transition that enhances the stem cell phenotype of GBM neurospheres and reduces sensitivity to TMZ treatment (Fig. 1h–k). We show, for the first time, that these reprogramming events require Oct4 and Sox2 direct binding to the promoter region of TGFBR2 to activate transcription by increasing chromatin accessibility (Fig. 2). Importantly, these stemness-driving molecular events are conserved during the transition of GBM cells to a more mesenchymal state (Fig. 2h–k), recapitulating key intermediate steps thought to result in GBM recurrence.^28,29^ Our current results also highlight miR-590-3p as a potent inhibitor of SMAD signaling that robustly blocked proliferation and self-renewal capacity of rGBM cells (Fig. 4d–f), similar to previous findings in glioma cell lines.^41,42^

As mentioned above, TGFβ signaling plays important roles in driving oncogenesis and blocking this pathway has shown promising anti-tumor activity.^1,2^ In GBM, previous approaches to block TGFβ signaling have encompassed a TGFBR1 small molecule inhibitor (galunisertib) and an ASO against TGFβ2 (trabedersen), both of which did not show improvements in overall survival in phase II trials.^43,44^ These efforts have mainly focused on targeting the TGFβ:TGFBR1 axis without much focus on either TGFBR2 or the complexity of downstream SMAD2/3 signaling and gene regulation. By integrating transcriptomic data from clinical rGBM specimens and miRNA target prediction algorithms we identified miR-590-3p as a broad inhibitor of multiple TGFBR2-associated SMAD2/3 targets (Fig. 4b). To translate these findings, we developed a novel PEGylated LiPBAE nanocarrier with low toxicity that efficiently distributes throughout an established tumor (Fig. 5j). Importantly, our novel PEGylated miR-590-3p nanomiRs reduce tumor size and extended survival of animals with seeming curative effects in a very aggressive orthotopic rGBM model (Fig. 6). These potent anti-tumor effects are concurrent with simultaneous reduction in expression of multiple putative oncogenes downstream of TGFBR2, essentially mimicking a combinatorial treatment strategy (Fig. 6), highlighting the translational potential of this approach.^45^

Approaches that focus on local drug delivery to the brain, such as convection-enhanced delivery (CED), are undergoing rapid improvement for long term drug delivery.^46^ Recent clinical trials have shown that prolonged CED (1 month) is safe for direct intratumoral delivery of chemotherapy,^47^ and additional work shows that large biomolecules, such as a recombinant poliovirus,^48^ can be delivered to CNS tumors via CED. The evolving landscape of chemical scaffolds for in vivo RNA delivery is providing new translational opportunities.^49^ We envision our nanomiR platform to be highly compatible with emerging CED-based approaches. Although our efforts highlight the use of local delivery using polymeric NPs, this multi-pronged approach leveraging the promiscuous nature of miRNA can be paired with other viral and non-viral nucleic acid delivery platforms to exploit tissue tropism for local delivery to other tumor types.^50^ This miRNA-based “top-down” approach allows us to provide new avenues for therapeutic intervention previously unavailable to circumvent the limitations in clinical success.

**Fig. 7:**
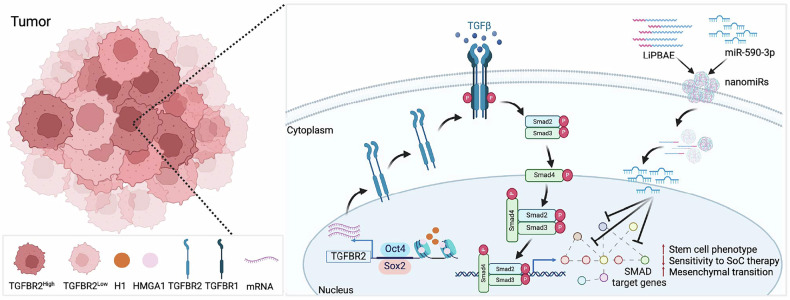
Proposed mechanistic model. GBM cells with high levels of TGFBR2 display enhanced SMAD2/3 signaling. These cells exhibit an accessible chromatin state at the TGFBR2 promoters associated with direct binding of architectural transcription factor HMGA1 and stem cell drivers Oct4 and Sox2. This increase in TGFBR2 levels associates with SMAD2/3 signaling activation, reduced sensitivity to TMZ and increases self-renewal capacity in GBM neurospheres. LiPBAE nanoparticles efficiently deliver miR-590-3p to simultaneously block multiple SMAD2/3 targets, induce tumor regression and prolong animal survival. These findings highlight the therapeutic potential of blocking TGFBR2 signaling in rGBM. Created in BioRender. Lopez-Bertoni, H. (2025) https://BioRender.com/u19d660

In summary, we show that TGFR2 plays important roles in determining the sensitivity of GBM cells to TMZ, the current standard-of-care therapeutic for GBM. Mechanistically, our work reveals that GBM cells with high levels of TGFBR2 display enhanced SMAD2/3 signaling associated with an accessible chromatin state at the TGFBR2 promoter. We demonstrate direct binding of architectural transcription factor HMGA1 and stem cell drivers Oct4 and Sox2 to the TGFBR2 promoter. We also show that blocking TGFBR2 can block self-renewal capacity and reduce sensitivity of GBM cells to TMZ. Additionally, we developed novel LiPBAE nanoparticles that efficiently deliver miR-590-3p to established rGBM human xenografts and we identified miR-590-3p as potent tumor suppressor that simultaneously block multiple SMAD2/3 targets, induce tumor regression, and prolong animal survival. These findings highlight the therapeutic potential of miR590-3p nanomiRs by blocking TGFBR2 signaling in rGBM (Fig. 7).

## Materials and Methods

### Cell culture

GBM-derived stem-like cells (GBM1A, GBM1B, M1123) are models of IDH wild-type GBM and were derived as described previously.^51,52^ The human GBM xenograft lines Mayo22 (IDH-wt, classical, treatment naïve), Mayo39 (IDH-wt, mesenchymal, treatment naïve), Mayo120 (IDH-wt, patients received radiation and TMZ) and Mayo192 (IDH-wt, patients received radiation, TMZ and Dasatinib) were obtained from the Mayo Clinic GBM Xenograft National Resource.^53^ The Mayo39R, Mayo22R, and GBM1AR cell lines were generated by exposing the parental lines to sub-lethal doses of IR (2GY) and TMZ and expanding the surviving fractions as described above. GBM neurospheres were grown using Stemline (R) Neural Stem Cell Expansion Medium (Sigma Millipore) supplemented with epidermal growth factor (20 ng/mL) and fibroblast growth factor (10 ng/mL). All cell lines used in the study were tested for mycoplasma and were short tandem repeat (STR) profiled.

For the extreme limiting dilution assays (ELDA), GBM cells were grown at decreasing cell densities ranging from 100 to 12.5 cells over 24 technical replicates for each dilution in conditions the enrich for stem-like cells, as described above. For experiments involving ITD1 treatment, either vehicle (dimethyl sulfoxide, DMSO) or ITD1 was added to the medium used for the master mix prior to dilution. For the experiments involving shRNAs, GBM cells first received lentivirus expressing the shRNA hairpin preceding dilution. Stem cell frequencies were determined 14 days after plating by counting the wells with spheres and inputting the results to the online ELDA tool (http://bioinf.wehi.edu.au/software/elda/).

### Lentivirus generation and cell transduction

The second-generation lentiviral packaging system from Addgene was used to generate lentivirus particles to transduce GBM1A (1 A) and GBM1B (1B) to generate stable transgenic lines expressing Oct4 and Sox2 (1A-O/S and 1B-O/S) or TGFBR2 (1A-R2 and 1B-R2) according to our established protocols.^15,30^ Plasmids for Oct4 (EX-Z0092-Lv156), Sox2 (EX-T2547-Lv160), and TGFBR2 (EX-Z4152-Lv242) were purchased from Gencopoeia and plasmids for short hairpin RNAs (shRNAs) against TGFBR2 were purchased from Millipore Sigma (TRCN0000194992 and TRCN0000040011).

### Luciferase assays

Genomic sequence corresponding to 2 kb upstream of the translation start site of TGFBR2 (NM_003242.6) was retrieved from the UCSC genome browser from assembly hg38 of the human genome. This sequence was used as input for transcription factor binding site prediction using the PROMO online tool (https://alggen.lsi.upc.es/cgi-bin/promo_v3/promo/promoinit.cgi?dirDB=TF_6.4) using transcription factor accession numbers T02368, T01836, T00651 and for HMGA1, Sox2 and Oct4 (POU5F1), respectively (Supplementary Fig. 3). Regions containing these binding sites were validated in our ChIP experiments and then cloned into the XhoI and BglII sites of the pGL4.2 vector (Promega), as previously described by us.^54^ The indicated reporter constructs were expressed in HEK293T and luciferase activity was measured 48 h after transfection using the Luciferase assay kit from Promega.

### LiPBAE synthesis

Bisphenol A glycerolate (1 glycerol/phenol) diacrylate (B7, Sigma Aldrich), 4-(2-Aminoethyl)morpholine (S90, Sigma Aldrich), 3-Morpholinopropylamine (S91, Sigma Aldrich), 1-dodecylamine (Sc12, Alfa Aesar), Oleylamine (Sc18, Sigma Aldrich), 1,3-diaminopropane (E1, Sigma Aldrich), 2-(3-Aminopropylamino)ethanol (E6, Sigma Aldrich), N,N-Dimethyldipropylenetriamine (E49, Sigma Aldrich), Diethylenetriamine (E63, EMD Millipore) were purchased and stored according to manufacturer’s instructions. Polymers were synthesized as described previously.^55^ Briefly, B7 monomer was combined with a hydrophilic sidechain monomer and a lipophilic sidechain monomer for a final concentration of 600 mg/mL (1.15:1 B7: sidechain ratio) in dimethylformamide and allowed to react for 48 h at 85 °C with stirring. The resulting base polymers were combined with an endcap monomer for a final concentration of 200 mg/mL in tetrahydrofuran and allowed to react for 2 h at 25 °C with stirring. Final polymers were precipitated and washed twice in diethyl ether, then dried under vacuum. Polymers were then resuspended in dimethyl sulfoxide at 100 mg/mL and stored at -20 °C with desiccant.

### In Vitro Transfection

For in vitro screening, Mayo120 and Mayo192 cells were plated in 96-well plates in 100 µL DMEM supplemented with 10% FBS and allowed to adhere overnight. Immediately prior to transfection, control Cy3 miRNA (Horizon Discovery Ltd) was diluted in 25 mM NaAc to a final miRNA concentration of 0.036 µg/µL and 7-90,c12-49 80% was diluted to a final concentration of 1.08 µg/µL. Equal volumes of dilute miRNA and polymer were combined, and nanomiRs self-assembled within minutes. NanomiRs were administered to cells for a final miRNA dose of 900 ng/mL and 27 µg/mL LiPBAE. Uptake, via Cy3, was assessed 24 h later via flow cytometry on an Attune NxT flow cytometer (ThermoFisher). aCSF was purchased from Biotechne (Catalog # 3525).

### In Vivo PEGylated NP Preparation

Control Cy3 miRNA or miR-590-3p (Horizon Discovery Ltd) was diluted in 3 M NaAc to a final miRNA concentration of 0.74 µg/µL. 7-90,c12-49 80% and DMG-PEG-2k were diluted in 100% ethanol to a final concentration of 30 µg/µL and 1.5 µg/µL, respectively. The ethanolic polymer/PEG was combined with the dilute miRNA, mixed vigorously, and PEGylated nanomiRs self-assembled within minutes. The mixture was then transferred to a 50 kDa MWCO dialysis device and was dialyzed against pH 7.5 PBS for 75 minutes at 4 °C. The final nanomiR volume was adjusted with PBS to a final miRNA concentration of 200 ng/µL and a final LiPBAE concentration of 6 µg/µL. NanomiRs were stored at -80 °C and thawed immediately before use.

### Immunoblotting and Chromatin immunoprecipitation

Western blots were performed by lysing cells in RIPA buffer (Sigma Millipore) for 30 min on ice and resolving equal amounts of protein (50–80 µg) by NOVEX 4–12% Tris-glycine gradient gel (Thermo Fisher Scientific). Samples were then transferred to nitrocellulose membranes (GE HealthCare) and blocked in LiCOR blocking buffer for 1 hour at room temperature before receiving primary antibodies. Secondary antibodies labeled with infrared dyes (LI-COR Biosciences) were used to detect the primary antibodies and protein levels were quantified using the Odyssey Infrared Imager (LI-COR Biosciences). Protein expression was then normalized to a house-keeping control.

The MAGnify Chromatin Immunoprecipitation System (Life Technologies, Grand Island, NY, USA), was used for chromatin immunoprecipitation assays following the manufacturer’s recommendations. Supplementary Table 1 and supplementary Table 2 lists the information for the antibodies and qRT-PCR primers used in this study, respectively.

### Intra-cranial nanomiR delivery and tumor formation in vivo

A transcranial cannula was placed so that the tip was in the right caudate/putamen of 8-week-old female NOD-SCID mice, as previously reported by us.^20^ Briefly, mice were anesthetized using a Ketamine (100 mg/kg)/ Xylazine (10 mg/kg) cocktail and mounted on a stereotactic frame. A rostro-caudal incision was made with a scalpel and a guide cannula (26 gauge) designed to have a Decon® mesh under the pedestal and cut 3 mm from the mesh was placed at coordinates: AP (antero-posterior) 0.0 (0 mm from bregma), L (lateral) 1.8 (1.8 mm right from mid-sagittal line). Seven days after cannula placement, animals received 2.0 × 10^4^ GBM cells via the cannula. Ten days after cell implantation, mice were randomly assigned to treatment groups and the control cohort received 5 µL PEGylated nanomiRs loaded with Cy3-labeled control miRNA and the experimental group received PEGylated nanomiRs loaded with miR-590-3p obtained from Horizon Discovery Ltd. In either group, each nanomiR infusion contained 1 µg miRNA and 30 µg LiPBAE in a 5 µL volume. Johns Hopkins Institutional Animal Care and Use Committee approved all animal procedures (Protocol# MO23M37) in accordance with the NIH Guide for the Care and Use of Laboratory Animals.

### Statistical and computational analyses

All data shown are representative of mean ± SD of triplicate results unless otherwise specified and all experiments were performed at least twice in each cell model (*N* ≥ 6). To compare multiple variables, we performed One- or Two-way analysis of variance (ANOVA) followed by the appropriate post hoc test (e.g. Tukey or Bonferroni). Single, two group comparisons were analyzed for variation and significance using a two-tailed, type 1 t-test. PRISM GraphPad 10 was used to perform all the statistical analyses presented and p values lower than 0.05 considered to be statistically significant.

RNA-seq and survival data from control and glioma patient was downloaded from the GlioVis database (http://gliovis.bioinfo.cnio.es/) and Betastasis (http://www.betastasis.com). Single nuclei RNA-Seq (snRNA-Seq) from recurrent GBM patients was retrieved from the Gene Expression Omnibus (GEO) data repository using accession number GSE174554. GSC scRNA-seq data was obtained from the Broad Institute Single Cell Portal (SCP503). scRNA-Seq data sets was analyzed using the Seurat R package as described below.

Gene ontology analysis was done using the gene set enrichment software (https://www.gsea-msigdb.org/gsea/index.jsp). Embryonic Stem cell signatures (M1871), SMAD2/3 (M2356), GBM Proneural (M2115), GBM Classical (M2121), GBM Mesenchymal (M2122) gene signatures were retrieved from the GSEA database. Additional GBM subtype gene signatures were obtained from Neftel et al.^11^

miRNA network analysis and target prediction was performed using the miRNet online platform (https://www.mirnet.ca/). A list of 158 genes enriched in rGBM shown to be SMAD2/3 targets (supplementary Table 3) was used as input for miRNA prediction using “human” as organisms and querying based on “gene symbols”. The results were then sorted based on “degree” to rank the results based on number of genes predicted to be downstream of each miRNA. The “mirnet_mir_target.csv” file containing the miRNA:gene pairs was used to retrieve the miR-590-3p targets.

### RNA-Seq, ATAC-Seq and Spatial transcriptomic analyses

RNA-Seq libraries were prepared by Novogene using their standard protocols and sequenced on an Illumina platform to generate paired-end reads. The reads were aligned to the reference genome hg38 and differential expression analysis experimental groups was determined using the DESeq2 R package (1.20.0). ATAC-Seq Libraries were constructed using the Buenrostro^56^ methodology. Briefly, 1.5 × 10^5^ freshly isolated nuclei were subject to Tn5 transposition at 37 °C for 30 min using the Nexterra workflow from Illumina. The purified DNA was then barcoded and amplified using a total of 16 PCR cycles. Libraries were then subject to paired-end sequencing, alignment, and differential expression analysis by Novogene as described above.

Spatial Transcriptomic analysis was performed on formalin-fixed paraffin embedded (FFPE) brain tissue sections derived from mice that received either control or miR-590-3p nanomiRs using the Visium CytAssist Spatial Gene Expression workflow from 10× genomics (Pleasanton, CA, USA). Briefly, the tumor-bearing tissue specimen was thinly sliced to 10 µm and CytAssist Visium was performed on a designated area of 6.5 mm × 6.5 mm square of a human transcriptome chip. The tissue was permeabilized and the mRNA was reverse-transcribed into cDNA with barcode containing slide location information. The Space Ranger pipeline v2022.0705.1 (10× Genomics, and the GRCh38-2020-A reference were used to process FASTQ files and generate Cell Ranger outputs. Data analysis was conducted using the Seurat R package (v4.4) in R Studio. Seurat objects were generated from count matrices generated from Cell Ranger outputs. Low quality cells, defined as number of features and/or counts < 500 and percentage of mitochondrial reads > 10%, were excluded and counts were normalized using the standard Seurat pipeline prior to downstream analyses. Dimensionality reduction was performed using the first 15 principal components and merged data sets from control and experimental groups were integrated and batched corrected using the Harmony algorithm. Expression scores for gene signatures were calculated using the Ucell R package and added as meta data to the Seurat Objects for further analysis. Differential gene expression analysis between the animals that received control and miR-590-3p nanomiRs was performed using the *do_ExpressionHeatmap* function of the SCpubr R package (v1.1.2). To measure gene expression differences within the tumors that received miR-590-3p nanomiRs, markers for the regions of interest were identified using the Loupe Browser from 10X genomics then used to assign identities to the clusters in the Seurat object. The *DotPlot* function was used to graph expression changes and number of cells expressing each gene.

## Supplementary information

Supplementary Material

Western Blots

Dataset 1

Dataset 2

Data set 4

Dataset 3

## Data Availability

The data supporting this study are available within the paper and its Supplementary Data file and via ArrayExpress collection in BioStudies Accession number: E-MTAB-14965.
