# Supplementary material for: Multipronged SMAD pathway targeting by lipophilic poly(β-amino ester) miR-590-3p nanomiRs inhibits mesenchymal glioblastoma growth and prolongs survival
Source: Signal Transduct Target Ther. 2025 Apr 30;10:145. doi: 10.1038/s41392-025-02223-w (PMC12041600; doi:10.1038/s41392-025-02223-w)
Supplement: Supplementary file 1 — Supplementary Material [file 41392_2025_2223_MOESM1_ESM.docx]

Supplementary Materials for

**Multipronged small mothers against decapentaplegic (SMAD) pathway targeting by lipophilic poly(β-amino ester) miR-590-3p nanomiRs inhibits mesenchymal glioblastoma growth and prolongs survival**

#Jack Korleski, #Sophie Sall, #Kathryn Luly, Maya K. Johnson, Amanda L. Johnson, Harmon Khela, Bachchu Lal, TC Taylor, Jean Micheal Ashby, Hector Alonso, Alice Li, Weiqiang Zhou, Karen Smith-Connor, Russell Hughes, Stephany Y. Tzeng, John Laterra, Jordan J. Green and Hernando Lopez-Bertoni.

**^#^**Authors contributed equally to this work

Correspondence to: Lopezbertoni@kennedykrieger.org

**This PDF file includes:**

Figures. S1 to S5

Tables S1 to S3

Figure. S1.


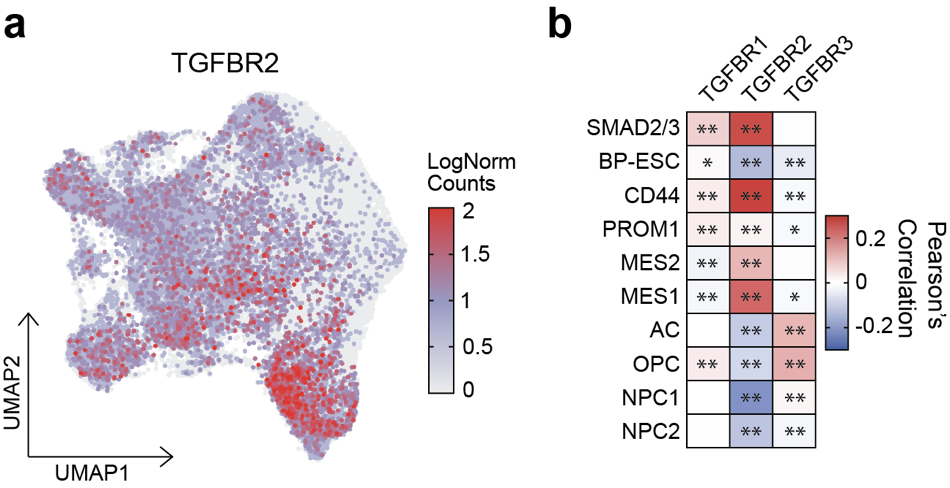


**Supplementary Figure 1: TGFβ receptor expression in GSCs. (a)** UMAP showing TGFBR2 expression in GSCs. **(b)** Heatmap showing Pearson’s correlations between TGFBR1, TGFBR2, TGFBR3 expression and markers or gene signatures related to GBM cell subsets determined from scRNA-Seq derived from GSCs.


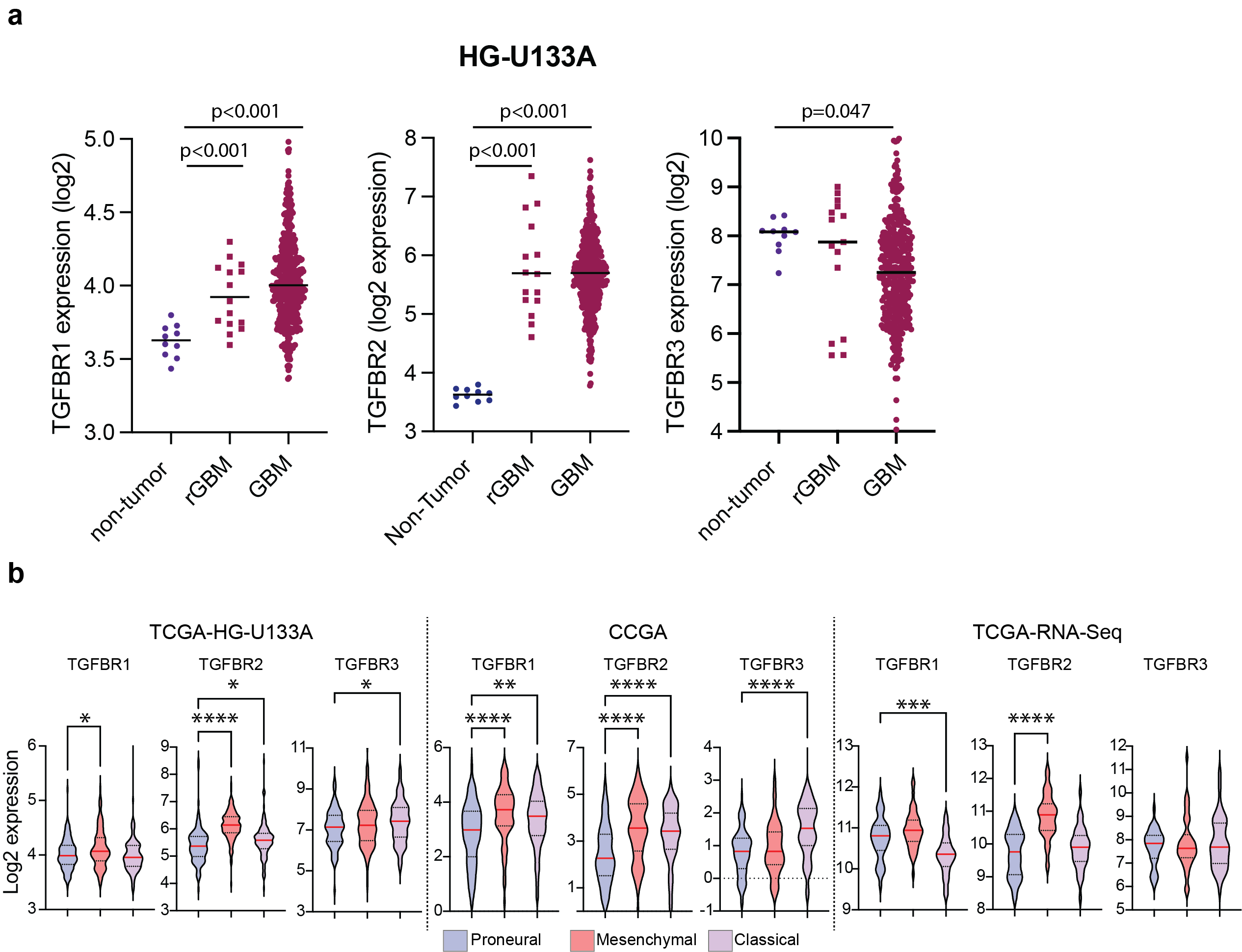
Figure. S2.

**Supplementary Figure 2: TGFβ receptor expression in GBM. (a)** TGFβ receptor expression in clinical specimens representing newly-diagnosed and recurrent GBM compared to normal brain. **(b)** Violin plots showing TGFBR1, TGFBR2, TGFBR3 expression in GBM stratified by molecular subtype. TCGA dataset HG-U133A was queried using the GlioVis portal. Statistical significance was calculated using One-way ANOVA with Dunnett's multiple comparisons test.

Figure. S3.

**TGFBR2 Promoter**

>hg38_knownGene_ENST00000295754.10 range=chr3:30605601-30691599 5'pad=0 3'pad=0 strand=+ repeatMasking=none

Oct4 (POU5F1) Sox2 (SOX2) HMGA1

ggaaagatacataatacctgccctcaagaaatttggagttgagtggaggatagaaatataaattaaagaatgacacaaataattataaagttacagctgttaaaagaaaagcatatggtgccaagagaacgtgtaatacaagatctactcatggaggtgagggaaagcttgcccatcaaagaagttatgattcaatccacgaagaccaggagttggctgggtgaagaaaaaaaggtcagaggaaggaagtccacactggggaaggctctaagcataaagggtaggaggattacagaggcatattcacgaaatttggagaaggctttcagtaagcaaggagaagccaaatgaaagtttacgggagagttggaggcttgaagacacgttcaaggatctggtttttatcttctctttatctcaagagcagtgggaagccattaaatgattttaatcagagggttggtataactagttttgtattttgaaaagctgaattcagctctcgtttgagaaactgagtgaaagagcccagaacggccgtggctgagggtgactcgtgggagactcctacacaagccatggcagtggcatgggctggtggcagaagagggaatagggagaagatttggaactcaatcttcctccattgacaaagtcactccagctttggcaaggcaattaattggtgggaaagaagatgcctagccctcctgatttcactgcactttctgcatcttcaacatgagtactgggaagtggcaaaacatccagaggcagcttgggtgctaggtggagcatgagttaaaattccaggatgaagcaaatgaacacttagaatgacaggaaagatttgggagttgggtttgggggagggctatttacctttattccctggagaccctggcacaaacccttgcctctgcaatcttcctctcaggtaaaggaattcattaaatgaattgctagaagatctactgaccagagggctgtacagaatcatatctttgagagtgggaagtaggttgatcacatagtttattatccaatcaggacatatctgaaagagaaagggggttctattaatatttaaactacaaaacatgtacaccaggaatgtcttgggcaaatctggttgccctagcaagaaaggaaatttgaaagtttatactgttctgctcccatgttaccccgtttgcacatgagagggtaagtattctctttcttcacctgcattaagggaataaaagcacaagcattcaggtgactcccaacccacttttaattttacagtttctgctatactctatacattctgaaaattacatttcccaccactatcacttcgtgataggtgatcatttacaattactcactgactcagtcccgggaagaggcggtgcaaaatgggacgctctatccaggtgctcattagaaatgcagaatctctgcctgcctcctagacctactgaattagaatctgcatttttaaataagatttccaggtgatcaatatgtacattaaaacttgagaaaaacctctagacttcgacctaaagaaaaacattttacaacttgacagtgtatgcacatacatacatgcatatagacacaactgaagcacaaatttaatgaagtagaatttaccgttactattttatttgggaaagaaatgtgctcgcgactcaatagattggagtattcactcctggatctcaacttgcaatttgaaaacgcatctctaaagcacctaggagcaatctgaagaaagctgaggggaggcggcagatgttctgatctactagggaaaacgtggacgttttctgttgttactttgtgaactgtgtgcacttagtcattcttgagtaaatacttggagcgaggaactcctgagtggtgtgggagggcggtgaggggcagctgaaagtcggccaaagctctcggaggggctggtctaggaaacatgattggcagctacgagagagctaggggctggacgtcgaggagagggagaaggctctcgggcggagagaggtcctgcccagctgttggcgaggagtttcctgtttcccccgcagcgctgagttgaagttgagtgagtcATGGGTCGGGGGCTGCTCAGGGGCCTGTGGCCGCTGCACATCGTCCTGTGGACGCGTATCGCCAGCACGATCCCACCGCACGTTCAGAAGTCGGTTAATAACGACATGATAGTCACTGACAACAACGGTGCAGTCAAGTTTCCACAACTGTGTAAATTTTGTGATGTGAGATTTTCCACCTGTGACAACCAGAAATCCTGCATGAGCAACTGCAGCATCACCTCCATCTGTGAGAAGCCACAGGAAGTCTGTGTGGCTGTATGGAGAAAGAATGACGAGAACATAACACTAGAGACAGTTTGCCATGACCCCAAGCTCCCCTACCATGACTTTATTCTGGAAGATGCTGCTTCTCCAAAGTGCATTATGAAGGAAAAAAAAAAGCCTGGTGAGACTTTCTTCATGTGTTCCTGTAGCTCTGATGAGTGCAATGACAACATCATCTTCTCAGAAGAATATAACACCAGCAATCCTGACTTGTTGCTAGTCATATTTCAAGTGACAGGCATCAGCCTCCTGCCACCACTGGGAGTTGCCATATCTGTCATCATCATCTTCTACTGCTACCGCGTTAACCGGCAGCAGAAGCTGAGTTCAACCTGGGAAACCGGCAAGACGCGGAAGCTCATGGAGTTCAGCGAGCACTGTGCCATCATCCTGGAAGATGACCGCTCTGACATCAGCTCCACGTGTGCCAACAACATCAACCACAACACAGAGCTGCTGCCCATTGAGCTGGACACCCTGGTGGGGAAAGGTCGCTTTGCTGAGGTCTATAAGGCCAAGCTGAAGCAGAACACTTCAGAGCAGTTTGAGACAGTGGCAGTCAAGATCTTTCCCTATGAGGAGTATGCCTCTTGGAAGACAGAGAAGGACATCTTCTCAGACATCAATCTGAAGCATGAGAACATACTCCAGTTCCTGACGGCTGAGGAGCGGAAGACGGAGTTGGGGAAACAATACTGGCTGATCACCGCCTTCCACGCCAAGGGCAACCTACAGGAGTACCTGACGCGGCATGTCATCAGCTGGGAGGACCTGCGCAAGCTGGGCAGCTCCCTCGCCCGGGGGATTGCTCACCTCCACAGTGATCACACTCCATGTGGGAGGCCCAAGATGCCCATCGTGCACAGGGACCTCAAGAGCTCCAATATCCTCGTGAAGAACGACCTAACCTGCTGCCTGTGTGACTTTGGGCTTTCCCTGCGTCTGGACCCTACTCTGTCTGTGGATGACCTGGCTAACAGTGGGCAGGTGGGAACTGCAAGATACATGGCTCCAGAAGTCCTAGAATCCAGGATGAATTTGGAGAATGTTGAGTCCTTCAAGCAGACCGATGTCTACTCCATGGCTCTGGTGCTCTGGGAAATGACATCTCGCTGTAATGCAGTGGGAGAAGTAAAAGATTATGAGCCTCCATTTGGTTCCAAGGTGCGGGAGCACCCCTGTGTCGAAAGCATGAAGGACAACGTGTTGAGAGATCGAGGGCGACCAGAAATTCCCAGCTTCTGGCTCAACCACCAGGGCATCCAGATGGTGTGTGAGACGTTGACTGAGTGCTGGGACCACGACCCAGAGGCCCGTCTCACAGCCCAGTGTGTGGCAGAACGCTTCAGTGAGCTGGAGCATCTGGACAGGCTCTCGGGGAGGAGCTGCTCGGAGGAGAAGATTCCTGAAGACGGCTCCCTAAACACTACCAAATAG

Supplementary Figure 3: TGFBR2 2Kb putative promoter region. Genomic sequence 2kB upstream of the TGFBR2 translation start site was retrieved from the USCS genome browser. Binding sites for Oct4 (blue boxes), Sox2 (red boxes), and HGMA1 (pink boxes) were determined using the PROMO web tool. Sequence in bold denotes the priming sites used for the ChIP experiments shown in Fig. 2b.

Figure S4:


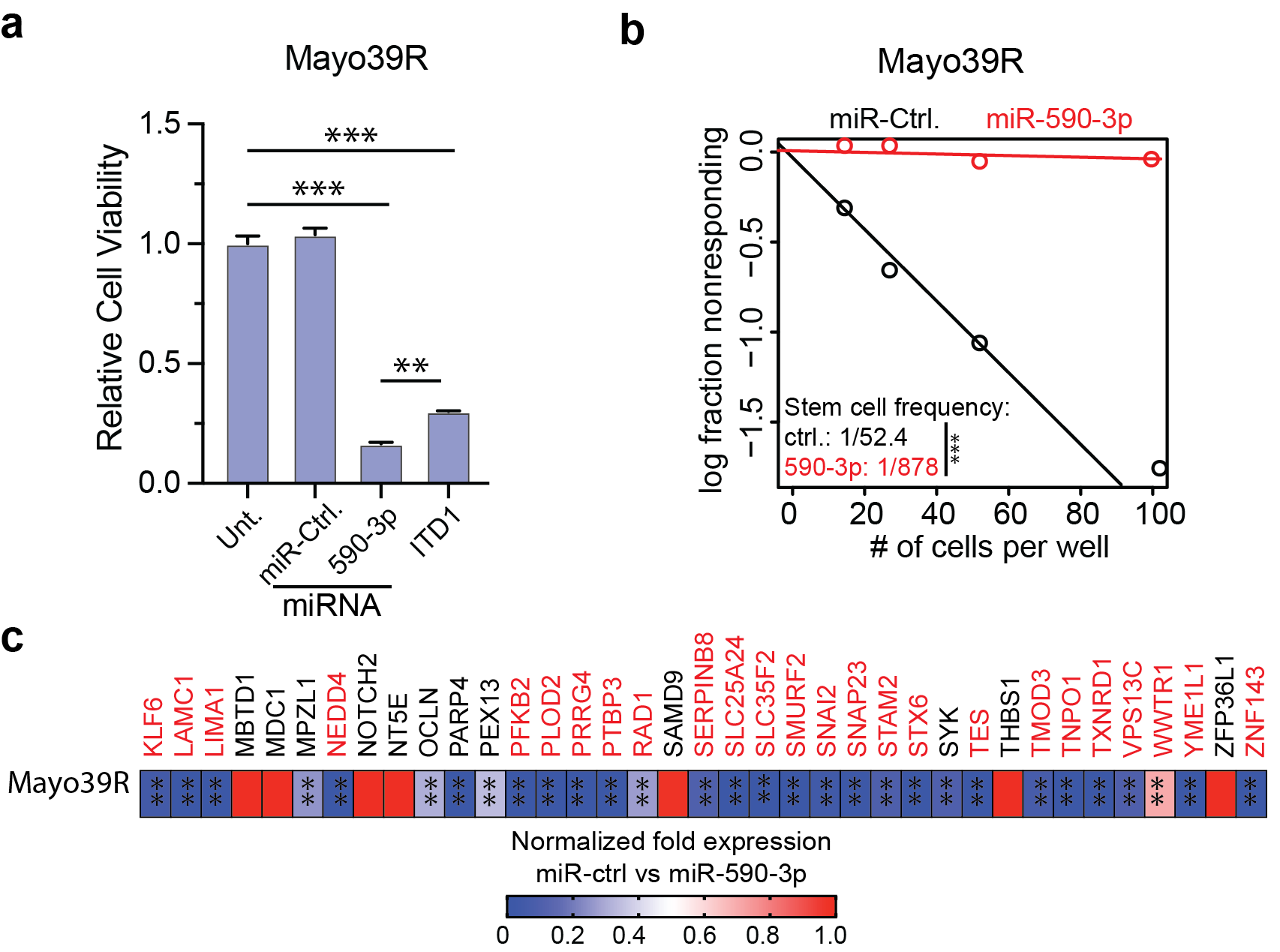


**Figure S4: miRNA-based targeting of TGFBR2 pathway inhibits the stem cell phenotype of therapy-resistant GBM cells. (a)** Cell viability 5 days after miR-590-3p or ITD1 treatment. **(b)** ELDA assay measuring stem cell frequency 14 days after transgenic miR-590-3p expression in TMZ-insensitive GBM cells.  **(c)** Expression of miR-590-3p predicted targets by qRT-PCR 5 days after transgenic miR-590-3p expression. Student T-test was used to determine statistical differences in panels **c**; One-way ANOVA with Tuckey's post hoc test was used to calculate statistical significance in panels **a**. Data are presented as means +/- S.D. **p<0.01; ***p<0.001


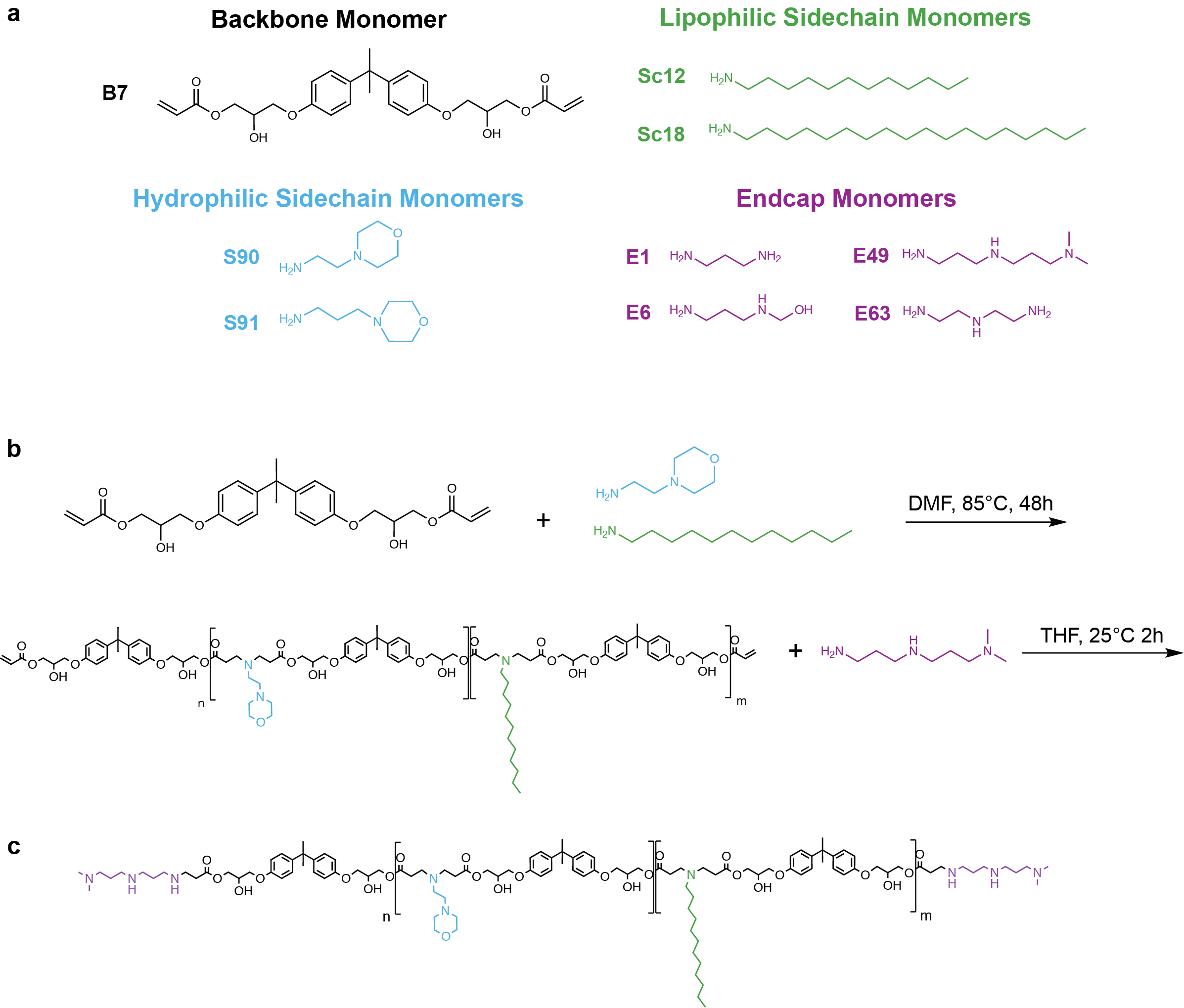
Figure S5:

**Figure S5: Structure and synthesis of LiPBAEs. (a)** Backbone, hydrophilic sidechain, lipophilic sidechain, and endcap monomers used. **(b)** Overview of polymer synthesis via two-step Michael addition. (**c)** Representative final polymer structure.

| **Supplementary table 1: Antibodies** | | | |
| --- | --- | --- | --- |
| **Western blots** | |  |  |
| **Target** | **Company** | **Catalog #** | **Dilution** |
| TGFBR2 | GENETEX | GTX129909 | 1:1,000 |
| pSMAD2 | Cell Signaling | 3108s | 1:1,000 |
| GAPDH | Santa Cruz | SC-47724 | 1:1,000 |
| **Chromatin Immuno-precipitation** | | |  |
| **Target** | **Company** | **Catalog #** | **Dilution** |
| Oct4 | Cell Signaling | 5677 | 1:100 |
| Sox2 | Cell Signaling | 2748 | 1:100 |
| HMGA1 | Cell Signaling | 7777 | 1:100 |
| IgG | Cell Signaling | 3900 | 1:100 |

| **Supplementary table 2: qRT-PCR primers** | | |
| --- | --- | --- |
| Stem cell and Lineage markers | | |
| **Target** | **Forward** | **Reverse** |
| 18S | ACA GGA TTG ACA GAT TGA TAG CTC | CAA ATC GCT CCA CCA ACT AAG AA |
| NESTIN | AAG ACT TCC CTC AGC TTT CAG | AGC AAA GAT CCA AGA CGC C |
| BMI1 | AAT CCC CAC CTG ATG TGT GT | GCT GGT CTC CAG GTA ACG AA |
| O4 | CTA CTG CTC TGG GTC CCA GG | CTG CCA CTG AAC CGA GAT GG |
| TUJ1 | CAA CAG CAC GGC CAT CCA GG | CTT GGG GCC CTG GGC CTC CGA |
| GFAP | GGC AAA AGC ACC AAA GAC GG | GGC GGC GTT CCA TTT ACA AT |
| SOX2 | GCC GAG TGG AAA CTT TTG TCG | GGC AGC GTG TAC TTA TCC TTC T |
| Oct4 | TGA ACT GTG GTG GAG AGT GC | AGG AAG GGC TAG GAC CAG AG |
| NANOG | CTA AGA GGT GGC AGA AAA ACA | CTG GTG GTA GGA AGA GTA AAG G |
| CD133 | AGT CGG AAA CT GCA GAT AGC | GGT AGT GTT GTA CTG GGC CAA T |
| CD44 | CTG CCG CTT TGC AGG TG TA | CAT TGT GGG CAA GGT GCT ATT |
| KLF4 | CCC ACA TGA AGC GAC TTC CC | CAG GTC CAG GAG ATC GTT GAA |
| OLIG2 | AAT ACC GTT ATG GAC TCG GAC GCC | ATT GTC GAC TCA CTT GGC GTC GGA |
|  |  |  |
| Chromatin Immunoprecipitation (ChIP) | | |
| **Target** | **Forward** | **Reverse** |
| TGFBR2_O/S | TCTCTGCCTGCCTCCTAGAC | AGTCGCGAGCACATTTCTTT |
|  |  |  |
| ATAC-qrPCR | | |
| **Target** | **Forward** | **Reverse** |
| TGFBR2 5' CNTRL | GTG CTC GCG ACT CAA TAG AT | CAG CTT TCT TCA GAT TGC TCC TA |
| TGFBR2 200bp | GTG AGG GGCAGC TGA AAG T | GAC TCA CTC AAC TTC AAC TCA GC |
|  |  |  |
| SMAD2/3 Targets | | |
| **Target** | **Forward** | **Reverse** |
| F11R | GTG CCT ACT CGG GCT TTT CTT | GTC ACC CGG TCC TCA TAG GAA |
| PDLIM1 | CCC AGC AGA TAG ACC TCC AG | TCT GAG CTT CCA AGT GTG TCA TA |
| SNAI2 | TGC GAT GCC CAG TCT AGA AA | AGA AAA AGG CTT CTC CCC CGT |
| TGFBR2 | GRA GCT CTG ATG AGT CGA ATG AC | CAG ATA TGG CAA CTC CCA GTG |
|  |  |  |
| miR-590-3p Targets | | |
| **Target** | **Forward** | **Reverse** |
| KLF6 | GACAGCTCCGAGGAACTTTCT | CACGCAACCCCACAGTTGA |
| LAMC1 | GGACTCCGCCCGAGGAATA | ACTTGAGACGCACATAGGTGA |
| LIMA1 | AACCAGCAGGTGTTTCACATC | TTGCTTGCCCATAGATCCTTG |
| MBTD1 | TGCAGTATCCTTTCAAACCTTGC | GCTTCGAGACCAACCAATATGA |
| MDC1 | GTAGGCCGAATGCCTGACTG | CGGAGGATAGGTGCCTTGTC |
| MPZL1 | ACGCCAAAAGAAATCTTCGTGG | TCAACCCGCCAGTCGTACTA |
| NEDD4 | CAGGCCCTCAATCACAAGC | AGGCCCTAGATCATTGGAAGT |
| NOTCH2 | CCTTCCACTGTGAGTGTCTGA | AGGTAGCATCATTCTGGCAGG |
| NT5E | GCCTGGGAGCTTACGATTTTG | TAGTGCCCTGGTACTGGTCG |
| OCLN | GACTTCAGGCAGCCTCGTTAC | GCCAGTTGTGTAGTCTGTCTCA |
| PARP4 | GTGAACAGGATTAGCCTCAACG | TCTTAGCCAATAGTCCCAGGTT |
| PEX13 | ACCTGGACAACCAGCACTTAC | GCCCAGCCCATTATATCCATAAC |
| PFKB2 | AGTCCTACGACTTCTTTCGGC | TCTCCTCAGTGAGATACGCCT |
| PLOD2 | TTATTGAGCAACCAACCCCTTT | GGCTTCCGCTTGACTTAGATTT |
| PRRG4 | CTGGTTCTACTCAGCCAACTG | GGTTGCCGGGAGTGAAGAG |
| PTBP3 | CCAGCCATTGGATTTCCTCAA | AAAAAGCCCATGTGGTGTGATA |
| RAD1 | CTATGCCAGGGACTTTAACTGC | AGGACTTCACTCGTCATATCCA |
| SAMD9 | GCAACCATCCATAGACCTGAC | AATAGTGCCATTGGTACGTGAAT |
| SERPINB8 | GCACTTTTGCCATCAGCTTATTT | CTCGGTGAATATCTCCGTCTTTG |
| SLC25A24 | GGTGCTGTCTCTCGAACAAG | CATCTGTCGAAAGCCACCAAA |
| SLC35F2 | TATGTGATCGTCAGAGCCTACC | TCCCTCCCTGCTAGTATGTCT |
| SMURF2 | GGCAATGCCATTCTACAGATACT | CAACCGAGAAATCCAGCACCT |
| SNAI2 | TGTGACAAGGAATATGTGAGCC | TGAGCCCTCAGATTTGACCTG |
| SNAP23 | ATGAGTCTCTGGAAAGTACGAGG | CCACAGCATTTGTTGAGTTCTG |
| STAM2 | GGAGCGAAAGATTGCCTAAAAGC | TCTGACCACTCCACCATTAAAGA |
| STX6 | CACCAACGAGCTGAGAAATAACC | CCCTGACAACTTGCCGAGT |
| SYK | TGCACTATCGCATCGACAAAG | CATTTCCCTGTGTGCCGATTT |
| TES | AGAGGATCGAAAAGTGGGAAAAC | CCTTGGGTAGCATCTGCATGTA |
| THBS1 | GCCATCCGCACTAACTACATT | TCCGTTGTGATAGCATAGGGG |
| TMOD3 | GTGACCTCGCAGCAATTCTTG | GTGGCTCATCAAATACCGGAA |
| TNPO1 | CTGTGAGGGAGCATTTGGTG | TGTGCAACATTAGAGCTTGAGTC |
| TXNRD1 | ATATGGCAAGAAGGTGATGGTCC | GGGCTTGTCCTAACAAAGCTG |
| VPS13C | ACTTGATAGTCTTAGCGCCTACT | ATCCAGTTTGGGCGTTTTGAG |
| WWTR1 | GATCCTGCCGGAGTCTTTCTT | CACGTCGTAGGACTGCTGG |
| YME1L1 | AGAATATAGCGCCATCATTCGTG | GGGTTCGCCTTAGGGAATCA |
| ZFP36L1 | ACTCCAGCCGCTACAAGAC | CGTAGGGGCAAAAGCCGAT |
| ZNF143 | AAGTCCCGCAGTCTGACAC | CCTGCTACACTTTCACTTCCATC |

| **Supplementary table 3: SMAD2/3 targets enriched in rGBM** | | | |
| --- | --- | --- | --- |
| UPP1 | PLAU | KRT15 | KLF3 |
| OCLN | SFN | TNC | TFAP2A |
| SGPL1 | PERP | TFPI2 | JUNB |
| KRT13 | S100A2 | TUBB6 | JUP |
| TNPO1 | KRT19 | THADA | NET1 |
| SMURF2 | TGIF1 | NFKB1 | RIN3 |
| NOTCH2 | RAB38 | THBS1 | VPS13C |
| MET | TES | PIP5K1A | ST7 |
| TRA2B | MCL1 | TNFRSF1A | MBTD1 |
| THBD | KRT7 | TRAM1 | PRSS22 |
| KCNJ15 | RIPK4 | WWTR1 | LGALS3 |
| TMCO3 | MPZL2 | PARP4 | LTBP3 |
| LIMK2 | LAMC2 | SERPINB7 | SRSF3 |
| PRKCH | SLC35F2 | PDLIM1 | MICALL1 |
| SMAD1 | MYC | SVIL | ZFP36L1 |
| KLF10 | TGFBI | KRT6A | KDM3A |
| RFC2 | ZBED2 | PNP | MCFD2 |
| TMEM87A | TM4SF1 | NEDD4 | PHLDA2 |
| SMC2 | WEE1 | LAD1 | MIOS |
| RMI1 | TNFRSF12A | SERPINB8 | PFKFB2 |
| MAP2K3 | SYNE2 | TSKU | PML |
| SMAD6 | PRSS23 | PLOD2 | REPIN1 |
| TRAPPC3 | YAP1 | PTGES | MDC1 |
| MDFI | TAGLN2 | SAMD9 | RIOK3 |
| STX6 | PLP2 | SLC25A24 | ZNF143 |
| PFN1 | KRT8 | ORC6 | METTL17 |
| TIPARP | SERPINB5 | PEX13 | LTBP2 |
| TXN | PLEK2 | STAM2 | SAT1 |
| KRT14 | SH2D4A | MMP10 | NT5E |
| POLR2J4 | MYOF | NXT1 | SYK |
| MMP9 | SLC4A2 | SNAP23 | PRRG4 |
| TXNRD1 | MALT1 | SNRPE | RAD1 |
| VCL | SNAI2 | STK10 | MAN1B1 |
| MTMR11 | PAWR | PTBP3 | PKP2 |
| MYH9 | KRT5 | TMOD3 | LAMC1 |
| ZW10 | THOC6 | MPZL1 | PPP1R13L |
| SAV1 | LIMA1 | MAP3K14 | KLF6 |
| SNX7 | RHOD | YME1L1 | TFAP2C |
| S100A10 | SERTAD2 | NFIL3 |  |
| MFSD12 | LAMB3 | PIM1 |  |
