# Supplementary figures and images for: Multipronged SMAD pathway targeting by lipophilic poly(β-amino ester) miR-590-3p nanomiRs inhibits mesenchymal glioblastoma growth and prolongs survival
Source: Signal Transduct Target Ther. 2025 Apr 30;10:145. doi: 10.1038/s41392-025-02223-w (PMC12041600; doi:10.1038/s41392-025-02223-w)

Figure 3a

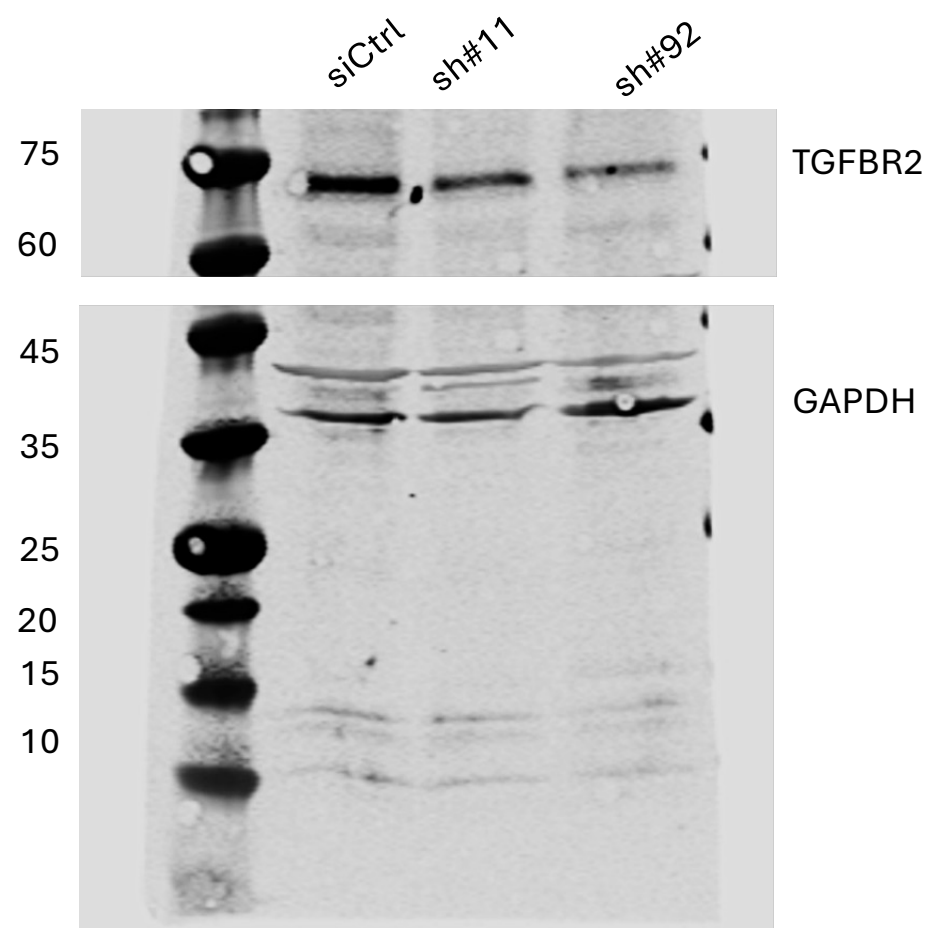

Figure 3c

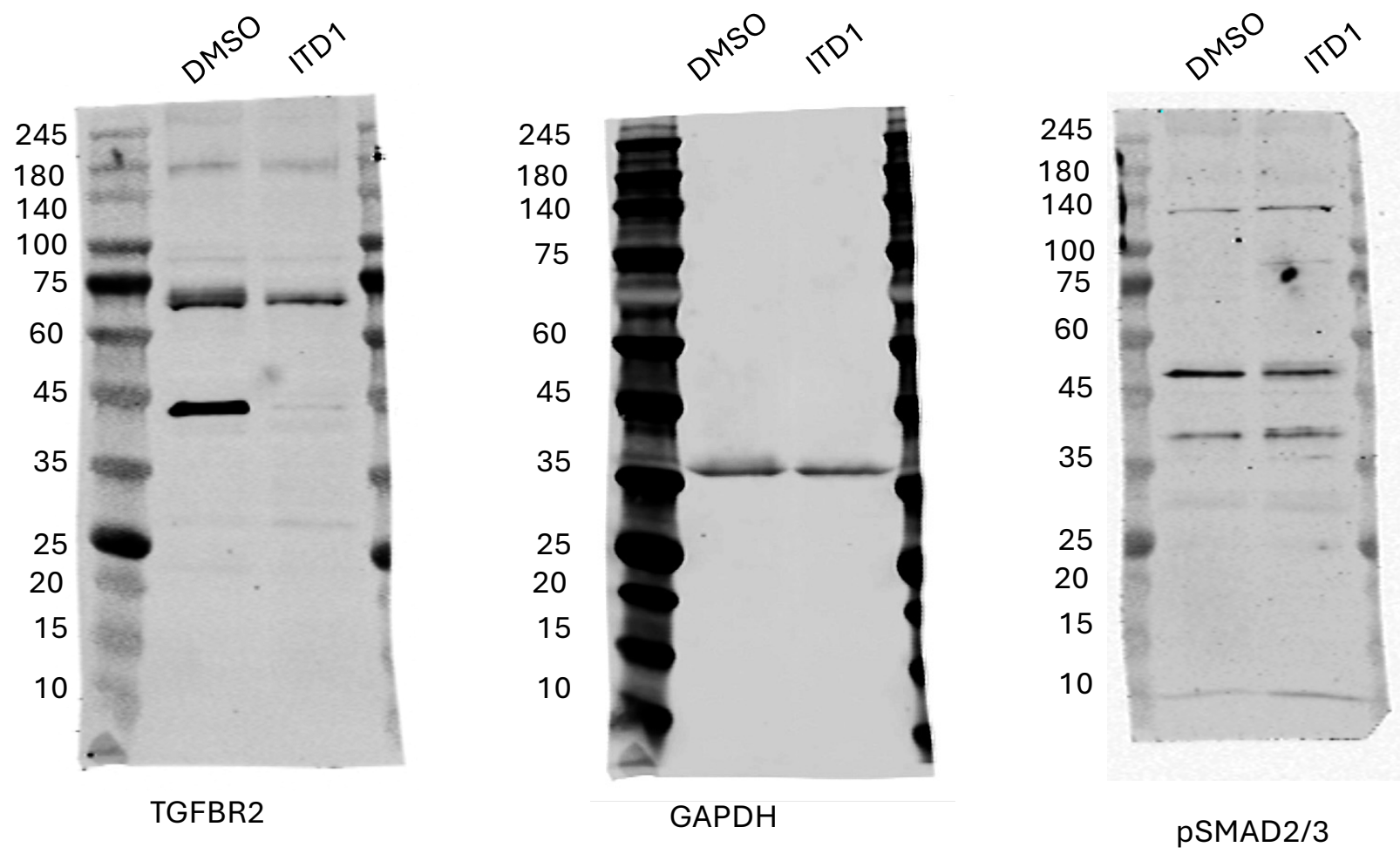

Supplement: Supplementary file 2 — Western Blots [file 41392_2025_2223_MOESM2_ESM.pdf]
